# Micro-level prediction of outstanding claim counts based on novel mixture models and neural networks

**DOI:** 10.1007/s13385-022-00314-4

**Published:** 2022-05-12

**Authors:** Axel Bücher, Alexander Rosenstock

**Affiliations:** 1 Mathematisches Institut, Heinrich-Heine-Universität Düsseldorf, Universitätsstraße 1, 40225 Düsseldorf, Germany; 2 ARAG SE, ARAG-Platz 1, 40472 Düsseldorf, Germany

**Keywords:** Loss reserving, Individual claim features, General insurance, Randomly truncated data, Expectation maximization algorithm, Mixture distribution

## Abstract

Predicting the number of outstanding claims (IBNR) is a central problem in actuarial loss reserving. Classical approaches like the Chain Ladder method rely on aggregating the available data in form of loss triangles, thereby wasting potentially useful additional claims information. A new approach based on a micro-level model for reporting delays involving neural networks is proposed. It is shown by extensive simulation experiments and an application to a large-scale real data set involving motor legal insurance claims that the new approach provides more accurate predictions in case of non-homogeneous portfolios.

**Supplementary Information:**

The online version contains supplementary material available at 10.1007/s13385-022-00314-4.

## Introduction

One of the classical challenges in non-life insurance consists of predicting parameters associated with outstanding claims, commonly referred to as IBNR claims for *incurred but not reported* [30]. Conventional approaches like the Chain Ladder method or the Bornhuetter-Ferguson method (see [33] for an introduction), which were proposed decades ago in view of the historic need for moderate computational costs, are based on aggregate claims data collected in so-called development triangles. Such an aggregation of claims data, however, is known to result in a huge loss of information, and likewise, possible computational restrictions became more and more superfluous due to the significant progress in technology. Therefore, many researchers have recently promoted the development of claims reserving methods that operate on individual data.

Many proposals regarding individual loss reserving rely on applications of celebrated Machine Learning (ML) techniques (see [18, 20] for general overviews), see, e.g.,  [8, 10–13, 28, 37, 38], among others. Most of the proposed methods have in common that they aim at modeling the development of each individual claim (in particular, each RBNS claim, for *reported but not settled*) and, if at all, use a Frequency-Severity or Chain Ladder based approach to estimate IBNR reserves over discrete time steps, usually one year. More precisely, [37] uses neural networks to obtain individualized Chain Ladder factors. Reference [12] uses neural networks to predict sets of aggregated IBNR run-off triangles. References [10, 38] model RBNS reserves using ML models and feature a Chain Ladder based approach to IBNR reserves. References [11, 28] focus completely on predicting RBNS reserves using ML models. Reference [8] applies tree based methods to both parts of the reserve.

The current paper contributes to this branch of the literature by proposing a new method to predict IBNR claim numbers. Our approach is based on a new flexible parametric model for the reporting delay distribution of an incurred claim, whose parameters are explained in terms of observed claims features by a classical multilayer perceptron neural network with multiple outputs.

The new parametric model, which might be of independent interest for general time-to-event modeling, builds upon a mixture construction proposed in [21] and involves a generalized Pareto tail, an Erlang mixture body and certain point measures. Statistical challenges to fit the model arise from the fact that observed reporting delays are subject to (random) truncation, which hampers a direct application of the classical EM algorithm [14] for mixture fitting based on (conditional) maximum likelihood (see [35] for fitting Erlang mixtures with non-random truncation). As a circumvent, we propose a suitable adaptation that relies on the ECME algorithm [27]; note that the ECME algorithm may exhibit faster convergence properties than the EM algorithm.

Estimation of the neural network parameters is done using TensorFlow, an industry-standard implementation framework for neural networks [1]. Optimization is carried out using the Adam and Nesterov-Accelerated Adam optimizers (see [15, 23], respectively) and a custom loss function is developed to adapt to the problem of fitting a parametric distribution to (randomly) truncated data. Starting values are provided by the global model fit based on the ECME-algorithm. Most implementation code is written using the R language and involves the keras and tensorflow R packages from [2, 9], respecively, as a binding to TensorFlow. The implementations are freely available as an R package called reservr on GitHub ([34]).

Finally, once the joint model for reporting delays has been fitted, we construct predictors for IBNR claim numbers based on a classical model for claims development involving a position-dependent marked Poisson processes, see [6, 31, 32]. Successful applications of this general idea can be found in [4, 5, 22], among others.

The new predictors are evaluated in a simulation study as well as in an application to a large-scale real-life dataset (about 250,000 contracts) concerning motor legal insurance claims. It is found that the new predictors outperform classical Chain Ladder approaches in simulation scenarios involving non-homogeneous portfolios and in the real-life example, with quite some advantage in the latter case.

The papers which are closest in spirit to the present approach are [4, 13]. The authors of the first paper concentrate on claim severities rather than claim numbers, and also use a neural network based approach for fitting semi-parametric distribution models of mixture type. A key difference to our approach is that the authors rely on three neural networks for modeling the distribution parameters, while our approach relies on only one neural network with multiple outputs. Additionally, we also face the challenge of (random) truncation, which is not present in the problem studied by [13]. On the other hand, [4] explicitly model reporting delays subject to (random) truncation using a parametric distribution. In contrast to our approach, they model small numbers of subgroups to allow more claim-level features to influence the distribution, which is close to our global approach used for finding suitable starting values for the neural network model.

The remaining parts of this paper are organized as follows. In Sect. 2, we start by summarizing the notation and then make some preliminary remarks on the integration of reporting delays into the classical position-dependent marked Poisson process model from [31]. We then construct both a new global model for reporting delays, with constant parameters not depending on individual claims features, and then a micro-level that incorporates the latter in terms of neural networks. Approaches to fit the models to (randomly) truncated data are presented in Sect. 3. The estimators may be transferred into predictors for IBNR claim counts, which is treated in Sect. 4. Results on a large-scale simulation study are presented in Sect. 5, and an application to a real dataset involving motor legal insurance claims is presented in Sect. 6.

## Modelling reporting delays

### Preliminaries on insurance portfolio data

Consider an insurance portfolio containing $N_\text {pol}$ independent risks. Each risk $\mathcal {P}$ is described by a coverage period $C = [t_{\text {start}}, t_{\text {end}}]$, and by risk features $\bar{x} \in \bar{\mathfrak {X}}$, where $\bar{\mathfrak {X}}$ is a feature space containing both discrete and continuous features; for example, information on the insured product and chosen options such as deductibles. Subsequently, we write $x = (C, \bar{x}) \in \mathfrak {X}= \{\text {intervals on } [0,\infty )\} \times \bar{\mathfrak {X}}$, and assume that *x* is constant over the course of the contract. In practice, risk features do change over time, but not very often, whence such a contract could be modelled as two separate risks.

Each risk can potentially incur claims during its coverage period, formally modelled by a claim arrival process. If a claim occurs at a (calendar) accident time $t_{\text {acc}} \in [t_\text {start}, t_{\text {end}}]$, it will not be immediately known to the insurer. The delay between accident time and time of reporting ($t_{\text {report}}$) results in incomplete information on the insurers side and thus necessitates the assessment of incurred but not yet reported (IBNR) claims. Of primal importance for any subsequent analysis (e.g., on cumulated claim sizes) is an accurate prediction of the number of IBNR claims, see below for details.

We view the reporting delay ($d_{\text {report}} :=t_{\text {report}} - t_{\text {acc}}$) as a mark on the claim arrival process. In addition to the reporting delay, there are several other claim features that are known to the insurer as soon as the claim is reported. We denote this feature space by $\mathfrak {Y}$. It will typically include information on the type of claim and maybe on its severity. The individual claim arrival processes, associated with the individual risks in the portfolio, are assumed to be (position-dependent) marked Poisson processes as in [31]. More precisely, following the notation in [24], we make the following assumption.

#### Model 1

(Claim Arrivals) Associated with each risk $\mathcal {P}^{(i)}$ in the portfolio, with risk features $x^{(i)}\in \mathfrak {X}$ among which we find the coverage period $C^{(i)}$, there is a position-dependent marked Poisson process with $N^{(i)} \sim {{\,\mathrm{Poi}\,}}\bigl (\int _{C^{(i)}} \lambda (x^{(i)}, t) \mathrm {\,d}t\bigr )$ points

$$\begin{aligned} \xi ^{(i)} = \sum _{j=1}^{N^{(i)}} \delta _{\left( T_{\mathrm {acc},j}^{(i)}, Y_{j}^{(i)}, D_{\mathrm {report}, j}^{(i)} \right) } \end{aligned}$$

on $$[0,\infty ) \times \mathfrak {Y}\times [0, \infty )$$ with:

(i) Intensity $\lambda (x^{(i)},t) \cdot \varvec{1}(t \in C^{(i)})$, i.e., for all intervals $[t_0, t_1] \subseteq [0,\infty )$, we have
  $$\begin{aligned} \sum _{j=1}^{N^{(i)}} \varvec{1}(T_{\mathrm {acc},j}^{(i)} \in [t_0, t_1]) = \int _{t_0}^{t_1} \xi ^{(i)}(\!\mathrm {\,d}t, \mathfrak {Y}, [0,\infty )) \sim {{\,\mathrm{Poi}\,}}\left( \int _{t_0}^{t_1} \varvec{1}(t \in C^{(i)}) \lambda (x^{(i)}, t) \mathrm {\,d}t \right) . \end{aligned}$$
(ii) Conditional claim feature distribution $P_Y(x^{(i)},t)=P_{Y \vert X=x^{(i)}, T_{\mathrm {acc}}=t}$. Here, *Y* denotes a generic claim feature variable containing all claim features except for the reporting delay, while *X* and $T_{\mathrm {acc}}$ are generic risk feature and accident time variables, respectively.
(iii) Conditional reporting delay distribution $P_D(x^{(i)}, t,y) = P_{D \vert X=x^{(i)}, T_{\mathrm {acc}}=t, Y=y}$. Here, $D=D_{\mathrm {report}}$ denotes a generic reporting delay variable, whose distribution is modelled conditional on the risk-claim variable $(X,T_{\mathrm {acc}}, Y)$.

Moreover, $\xi ^{(1)}, \dots , \xi ^{(N_\mathrm {pol})}$ are mutually independent.

Note that the overall intensity measure of $\xi ^{(i)}$ can be written as

$$\begin{aligned} \mu ^{(i)}(A) = \int _{C^{(i)}} \int _{\mathfrak {Y}} \int _{[0, \infty )} \varvec{1}((t,y,d) \in A) \lambda \left( x^{(i)}, t\right) P_D\left( x^{(i)}, t,y\right) (\!\mathrm {\,d}d) P_Y\left( x^{(i)},t\right) (\!\mathrm {\,d}y) \mathrm {\,d}t. \end{aligned}$$

This paper is mainly concerned with the reporting delay $D_{\mathrm {report}}$. More precisely, in the subsequent sections, we will propose (1) a parametric model for $P_D$ that is both flexible and analytically tractable (Sects. 2.2 and 2.3), and (2) an estimation approach for the model that adequately takes care of the major nuisance that available observations are typically randomly right-truncated (Sect. 3). We impose the following assumption on the data-generating process.

#### Observation Scheme 1

At given calendar time $\tau$, the available dataset $\mathfrak {D}=\mathfrak {D}_\tau$ consists of all risk features $x^{(i)}$, $i\in \{1, \dots , N_{\text {pol}}\}$, and all reported claim data up to calendar time $\tau$, i.e.

1 $$\begin{aligned} \bigl \{ (x^{(i)}, t_{\mathrm {acc},j}^{(i)}, y_{j}^{(i)},d_{\mathrm {report},j}^{(i)}) \,\big \vert \, t_{\mathrm {report}, j}^{(i)} :=d_{\mathrm {report},j}^{(i)} + t_{\mathrm {acc},j}^{(i)} \le \tau \bigr \}. \end{aligned}$$

Equivalently, we observe, for each $i\in \{1, \dots , N_{\text {pol}}\}$, the risk feature $x^{(i)}$ and the restriction $\xi ^{(i)}_r(\cdot ) = \xi ^{(i)}(\, \cdot \, \cap R_{\tau })$, where $R_\tau = \{(t,y,d): d+t \le \tau \}$ and where the lower index *r* stands for ‘*reported*’. Note that the observations in (1) are randomly right-truncated, which requires additional care when estimating the model.

Note that the ultimate objective of claims reserving is to obtain good (aggregate) predictions for characteristics that depend on the partly unobserved paths of $\xi ^{(i)}$ across different time and feature sections, based on reported observations $\xi ^{(i)}_r$ as in Observation Scheme 1. Details are provided in Sect. 4, where explicit predictors are derived that depend on the (fitted) reporting delay models described in the next two sections.

### A Global Parametric Model based on Blended Distributions

Reporting delays exhibit some stylized facts that appear to be present in many empirical data sets:

- First, in the *lower tail*, they are non-negative with very short reporting delays (such as $0,1,2, \dots$ days) being quite common. Short reporting delays may further be influenced by certain calendar effects (e.g., across weekends), whence rather flexible models are needed for the lower tail.
- On an intermediate timescale (the *body of the distribution*), reporting delays can be considered quasi-continuous and only exhibit small specific patterns. However, the general shape of the distribution differs significantly between clusters of similar claims, suggesting the use of mixture type models for large heterogeneous portfolios.
- Finally, in the *upper tail*, very long reporting delays may exist depending on the line of business, suggesting some heavy tailed behaviour.

Models for each of the three parts of the distribution are described below, to be merged later into an appropriate mixture model.

First, in the interest of maximizing flexibility, we propose to model the discrete lower tail by a mixture of Dirac-components (see also [4]), i.e., by $\sum _{i=1}^n p_i^{(\delta )} \delta _{i-1}$, where $\delta _i$ denotes the Dirac measure at *i*, where $p_i^{(\delta )}$ are mixture weights, and where the choice of *n* is driven by a case-specific analysis of the data, a reasonable starting value being $n = 8$ corresponding to one week.

Next, consider modeling the body of the reporting delay distribution. A good choice for a flexible continuous model is provided by a (translated) Erlang Mixture, because the latter family is dense in the space of positive distributions with respect to weak convergence [26], and hence provides sufficient flexibility for adapting to real-life distributions. For combining (mixing) the Erlang Mixture component with the discrete lower tail, we propose to translate the Erlang Mixture component by $n - \tfrac{1}{2}$ such that its support does not intersect with the discrete components but additionally the smallest possible observation that does not belong to the discrete components, namely $d_{\text {report}} = n$, is in the interior of the support of the continuous component. If we translated by *n* or $n - 1$ instead, observations from the data would touch the boundary of the support, leading to numerical instability.

Next, consider the tail model, whose need is motivated by the fact that the tail behaviour of Erlang Mixtures is, as they are mixtures of Gamma Distributions, fixed to exponential decay (i.e., the extreme value index is 0, see [16]). In order to better capture possible heavy tail behaviour, we chose to attach to the Erlang Mixture body a Generalized Pareto Distribution with non-negative shape parameter. The latter family satisfies our need for flexibility in the heaviness of the tail and for a parsimonious parametrization, and may further be motivated by the Pickands-Balkema-de Haan theorem ([16]). Recall that the Generalized Pareto Distribution ${{\,\mathrm{GPD}\,}}(\mu , \sigma , \xi )$ has cumulative distribution function (cdf)

2 $$\begin{aligned} G_{\mu , \sigma , \xi }(x) = 1 - \Big (1 + \xi \frac{x - \mu }{\sigma }\Big )^{-1/\xi }, \quad x \ge \mu , \end{aligned}$$

with parameters $\mu \in \mathbb {R}$ (location), $\sigma >0$ (scale), and $\xi \ge 0$ (shape). Practically the reporting delay should have finite expectation, so we constrain the GPD component to have shape parameter $0 \le \xi < 1$, where $\xi = 0$ degenerates to an Exponential distribution which is also a member of the Erlang Mixtures.

Classical approaches of attaching a heavy-tailed distribution to a body distribution use hard cut-off thresholds that result in jump discontinuities in the resulting density. This jump can be avoided at little extra computational cost by using what we call *blended distributions* below. The construction goes back to [21, Section 2], and relies on gradually mixing two cumulative distribution functions in a blending interval *A* centered at some (high) threshold $\kappa$, eventually yielding a smooth density. We follow up on their ideas but, in the interest of increased flexibility, allow $\kappa$ to be a parameter of the family instead of being determined by the blended component distributions.

#### Definition 1

(*Blended Distribution family*) Given two distributions *P*, *Q* on $\mathbb {R}$ with cdfs $F(\cdot )=P((-\infty , \cdot ])$ and $G(\cdot )=Q((-\infty , \cdot ])$, respectively, and parameters $\kappa \in \mathbb {R}, \varepsilon \in \mathbb {R}_+, p \in [0, 1]^2, p_1 + p_2 = 1$ such that $F(\kappa ) >0$ and $G(\kappa ) < 1$, we define the *Blended Distribution* $B = {{\,\mathrm{Blended}\,}}(P, Q; p, \kappa , \varepsilon )$ of *P* and *Q* with blending interval $[\kappa - \varepsilon , \kappa + \varepsilon ]$ and mixture probabilities *p* via its cdf $F_B$:

3 $$\begin{aligned} p_{\kappa , \varepsilon }(x)&= {\left\{ \begin{array}{ll} x &{} , x \in (-\infty , \kappa -\varepsilon ],\\ \tfrac{1}{2} (x + \kappa - \varepsilon ) + \tfrac{\varepsilon }{\pi }\cos \Big ( \frac{\pi (x - \kappa )}{2 \varepsilon } \Big ) &{}, x \in (\kappa -\varepsilon , \kappa +\varepsilon ], \\ \kappa &{}, x \in (\kappa +\varepsilon , \infty ), \end{array}\right. } \nonumber \\ q_{\kappa , \varepsilon }(x)&= {\left\{ \begin{array}{ll} \kappa &{} , x \in (-\infty , \kappa -\varepsilon ],\\ \tfrac{1}{2} (x + \kappa + \varepsilon ) - \tfrac{\varepsilon }{\pi }\cos \Big ( \frac{\pi (x - \kappa )}{2 \varepsilon } \Big ) &{}, x \in (\kappa -\varepsilon , \kappa +\varepsilon ], \\ x &{}, x \in (\kappa +\varepsilon , \infty ), \end{array}\right. } \nonumber \\ F_B(x)&= p_1 \frac{F(p_{\kappa , \varepsilon }(x))}{F(\kappa )} + p_2 \frac{G(q_{\kappa , \varepsilon }(x)) - G(\kappa )}{1 - G(\kappa )}. \end{aligned}$$

**Fig. 1:**
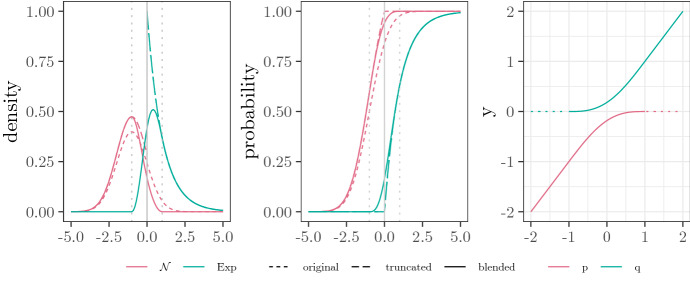
Illustration of the mixture components in relation to the original families for ${{\,\mathrm{Blended}\,}}(\mathcal {N}(-1,1), {{\,\mathrm{Exp}\,}}(1); 0, 1)$. Depicted are the density and the cdf from the original, plain truncated and blended component distributions. Plain truncation refers to truncation from above at $\kappa = 0$ for $\mathcal {N}(-1, 1)$ and truncation from below at $\kappa = 0$ for ${{\,\mathrm{Exp}\,}}(1)$; note that the latter coincides with the original ${{\,\mathrm{Exp}\,}}(1)$ distribution. The right panel shows the corresponding blending functions, $p_{\kappa , \varepsilon }$ and $q_{\kappa , \varepsilon }$. Irrelevant regions, where the corresponding components have no mass, are dotted. Compare [21, Figure 1]

See the right panel in Fig. 1 for the graph of $p_{\kappa , \varepsilon }$ and $q_{\kappa , \varepsilon }$.

Given two families $\mathcal {F}, \mathcal {G}$ of distributions on $\mathbb {R}$, and parameters $\kappa \in \mathbb {R}, \varepsilon \in \mathbb {R}_+$ (where $\mathcal {F}$ or $\mathcal {G}$ are allowed to depend on $\kappa$ and $\varepsilon$), we define the *Blended Distribution family* as the family of Distributions

4 $$\begin{aligned} {{\,\mathrm{Blended}\,}}(\mathcal {F}, \mathcal {G}; \kappa , \varepsilon )&:=\{ {{\,\mathrm{Blended}\,}}(P, Q ; p, \kappa , \varepsilon ) \mid P \in \mathcal {F}, Q \in \mathcal {G}, p \in [0, 1]^2, \Vert p\Vert _1 = 1 \}. \end{aligned}$$

Note that $F_B$ defined in (3) is a mixture of two distributions, say $P'$ and $Q'$, that are obtained from a certain truncation-like transformation applied to input distributions *P* and *Q*, respectively, in such a way that $P'$ is supported on a subset of $(-\infty , \kappa +\varepsilon ]$, while $Q'$ is supported on a subset of $[\kappa -\varepsilon , \infty )$. The transformed cdfs and densities are illustrated for $P = \mathcal {N}(-1,1)$ and $Q = {{\,\mathrm{Exp}\,}}(1)$ in Fig. 1, alongside with respective curves for the distributions obtained by plain upper or lower truncation at $\kappa$. Note that, in practice, the choice of a suitable blending region defined by $\kappa$ and $\varepsilon$ is similar to the choice of the cut-off threshold in conventional tail modelling problems. Throughout the applications in this paper, we experimented with blending regions that are defined by different empirical quantiles close to 1. By doing so, we eventually control the number of observations used for fitting the tail.

If the families $\mathcal {F}$ and $\mathcal {G}$ in (4) are parameterized by sets $\Theta _F$ and $\Theta _G$, then the mixture component families making up ${{\,\mathrm{Blended}\,}}(\mathcal {F}, \mathcal {G}; \kappa , \varepsilon )$ are defined by their cdfs

5 $$\begin{aligned} \mathcal {F}'&= \Bigl \{ F' \Bigm \vert F' = \frac{F \circ p_{\kappa ,\varepsilon }}{F(\kappa )} \text { for some } F \in \mathcal {F}\Bigr \}, \end{aligned}$$

6 $$\begin{aligned} \mathcal {G}'&= \Bigl \{ G' \Bigm \vert G' = \frac{G \circ q_{\kappa ,\varepsilon } - G(\kappa )}{1 - G(\kappa )} \text { for some } G \in \mathcal {G}\Bigr \}, \end{aligned}$$

which are naturally parameterized by the same parameter space. Care must be taken to preserve identifiability of the parametrization as ‘$\theta _1 \ne \theta _2 \Rightarrow F_{\theta _1} \ne F_{\theta _2}$’ does not necessarily imply the same property for $\mathcal {F}'$. For an example where this in not the case, consider the family of uniform distributions $\mathcal {U}= \{U_b = \mathrm {Unif}(0, b): b \in \Theta _{\mathcal {U}} = (0, \infty ) \}$. If taken as the left side ($\mathcal {F}$) of a blended distribution, the blended components $U_b'$ will be the same distribution for all $b \ge \kappa$. Note that a simple sufficient condition for identifiability is $\bigcup _{\theta \in \Theta _F} {{\,\mathrm{supp}\,}}\mathcal {F}_{\theta } \subseteq (-\infty , \kappa ]$ and $\bigcup _{\theta \in \Theta _G} {{\,\mathrm{supp}\,}}\mathcal {G}_{\theta } \subseteq [\kappa , \infty )$.

The final distribution model that we employ for modelling reporting delays is as follows.

#### Definition 2

(*Blended Dirac-Erlang-Generalized Pareto family*) Given parameters $n, m \in \mathbb {N}_0$, and $\kappa , \varepsilon \in (0,\infty )$, we define the *Blended Dirac-Erlang-Generalized Pareto family* as the family of Distributions

$$\begin{aligned} &{{\,\mathrm{BDEGP}\,}}(n, m, \kappa , \varepsilon ) \\ &:=\Bigl \{ \sum _{i = 1}^n p_i^{(\delta )} \delta _{i - 1} + p^{(\delta )}_{n + 1} {{\,\mathrm{Blended}\,}}\Big ( \sum _{i = 1}^m p^{(e)}_i (\Gamma _{\alpha _i, \theta } + n - \tfrac{1}{2}), {{\,\mathrm{GPD}\,}}_{\mu = \kappa , \sigma , \xi }; p^{(b)}, \kappa , \varepsilon \Big ) \\& \Bigm \vert p^{(\delta )} \in [0, 1]^{n + 1}, p^{(e)} \in [0, 1]^m, p^{(b)} \in [0, 1]^2, \\& \sum p^{(\delta )}_i = \sum p^{(e)}_i = p_1^{(b)} + p_2^{(b)} = 1, \\& \alpha \in \mathbb {N}^m, \alpha _1< \dots < \alpha _m, \theta \in \mathbb {R}_+, \sigma \in \mathbb {R}_+, \xi \in [0, 1) \Bigr \}. \end{aligned}$$

**Fig. 2:**
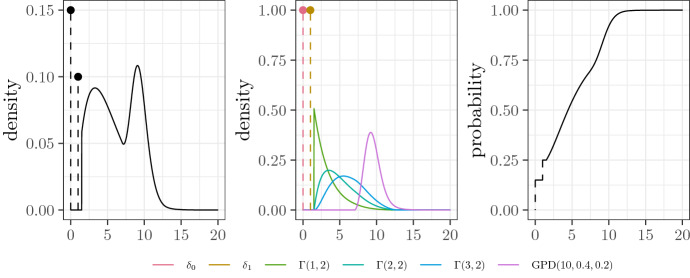
${{\,\mathrm{BDEGP}\,}}(2, 3, 10, 3)$ distribution. Parameters: $p^{(\delta )} = (0.15, 0.1, 0.75)$, $p^{(b)} = (0.7, 0.3)$, $p^{(e)} = (0.2, 0.5, 0.3)$, $\theta = 2$, $\alpha = (1, 2, 3)$, $\sigma = 0.4$, $\xi = 0.2$. left: density, middle: component densities, right: cdf. Note how the component densities are smoothed over (7, 13) in comparison to truncated Erlang distributions or ${{\,\mathrm{GPD}\,}}_{\mu = 10, \sigma = 0.4, \xi = 0.2}$

A specific distribution from ${{\,\mathrm{BDEGP}\,}}(2, 3, 10, 3)$ is illustrated in Fig. 2.

This distribution family has $2m + n + 3$ degrees of freedom due to the constraints placed on the mixture parameters $p^{(\delta )}, p^{(e)}$, and $p^{(b)}$. Note that due to the restriction of $\xi < 1$, all members of this family are guaranteed to have finite expectation, though higher moments may not exist.

Returning to the context of Sect. 2, in a simplified parametric global model we assume that, for some fixed hyperparameters *n*, *m*, $\kappa$, and $\epsilon$, the reporting delay distribution for each claim lies in ${{\,\mathrm{BDEGP}\,}}(n, m, \kappa , \epsilon )$. Here, ‘global’ refers to the fact the reporting delay distribution does not depend on accident time or risk and claim features.

#### Model 2

(Parametric Global Model) Next to the assumptions made in Model 1 assume that, for some given (known) parameters $n, m, \kappa$, and $\varepsilon$, we have

7 $$\begin{aligned} P_D(x, t, y) \equiv B_{\tilde{\theta }} \qquad \text { for all } x,t,y \end{aligned}$$

for some $B_{\tilde{\theta }}\in {{\,\mathrm{BDEGP}\,}}(n, m, \kappa , \varepsilon )$. Here, all free parameters of the ${{\,\mathrm{BDEGP}\,}}(n, m, \kappa , \epsilon )$-model are collected in a vector $\tilde{\theta }= (p^{(\delta )}, p^{(e)}, p^{(b)}, \alpha , \theta , \sigma , \xi )$ with respective parameter space $\Theta \subset [0, 1]^{n + 1} \times [0, 1]^m \times [0, 1]^2 \times \mathbb {N}^m \times \mathbb {R}_+ \times \mathbb {R}_+ \times [0, 1) \subset \mathbb {R}^{2\,m + n + 6}$ with effective dimension $2m + n + 3$.

Despite its simplicity, the global model will prove useful for finding good starting values for a fitting algorithm for the micro-level model introduced next.

### A micro-level model based on neural networks

Quite naturally, the micro-level model is based on an extension of the global model by allowing $\tilde{\theta }= g(x, t, y)$ to depend on claim and risk features. More precisely, we assume that *g* is a neural network of some predefined architecture.

#### Model 3

(Micro-Level Model) Next to the assumptions made in Model 1 assume that, for some given (known) parameters $n, m, \kappa$, and $\varepsilon$, we have

$$\begin{aligned} P_D(x, t, y) \equiv B_{g(x, t, y)} \qquad \text { for all } x, t, y \end{aligned}$$

for some $g \in \mathcal {G}$, where $\mathcal {G}$ denotes a set of neural networks $g: \mathfrak {X}\times \mathbb {R}\times \mathfrak {Y}\rightarrow \Theta$ such that $B_{g(x, t, y)} \in {{\,\mathrm{BDEGP}\,}}(n, m, \kappa , \varepsilon )$ for all $(x, t, y) \in {{\,\mathrm{dom}\,}}(g)$.

#### Remark 1

Instead of postulating $\mathcal {G}$ to be a family of neural networks, it is also possible to consider alternative functional relationships $g: \mathfrak {X}\times \mathbb {R}\times \mathfrak {Y}\rightarrow \Theta$. For the sake of brevity, we limit ourselves to neural networks in this paper, which have proven useful in numerous applications due to their great flexibility and the efficient fitting algorithms. Likewise, neural networks may be combined with other parametric global models such as the Dirac-Weibull-mixture model from [4]. The latter was found to provide less efficient predictors in preliminary experiments, whence we restrict attention to the BDEGP family.

It remains to explain the class of neural networks $\mathcal {G}$; see [18] for a good introduction to neural networks. We chose a classical multilayer perceptron (MLP) neural network with $N_\text {dense}$ hidden layers of dimension $n_1, \ldots , n_{N_{\text {dense}}}$. Discrete data were incorporated using embedding layers, and the final dense layer was mapped to the parameter space $\Theta$ via canonical transformations (softmax for probability weights, softplus for positive parameters, sigmoid for interval-bounded parameters, and identity for unbounded parameters). We call this canonical mapping $f_{\text {adaptor}}: \mathbb {R}^{n_{\text {tail}}} \rightarrow \Theta$ where $n_{\text {tail}}$ is the output dimension of the final dense layer. A more detailed description of the neural network architecture can be found in Appendix A in the supplementary material.

The neural network construction is not valid for integer components in $\Theta$. For this reason, we must fix the shape parameters of the erlang components in the micro-level BDEGP-model. In the interest of maximizing flexibility, one could argue to fix the shapes to $1, \ldots , M$ for some large integer *M*, such that $\mathcal {F}$ contains all erlang mixtures with shapes at most *M*. However, this heuristically results in overparametrization, whence we propose to fix the shapes to the values obtained from estimating the global model instead, say $\alpha =(\alpha _1, \dots , \alpha _m)$. In addition to that, we have found the parameter $\xi$, although real-valued, to pose numerical challenges. Individual-level parameter estimates of $\xi$ quickly converged to 1 leading to poor performance and instability. Therefore, $\xi$ was replaced by the (fixed) initial value obtained from fitting Model 2. Formally, this means that ${{\,\mathrm{BDEGP}\,}}(n, m, \kappa , \varepsilon )$ in Model 3 will be replaced by

$$\begin{aligned} &{{{\,\mathrm{BDEGP}\,}}}_{\text {fix}}(n, m, \kappa , \varepsilon , \alpha , \xi ) \\ &:=\Bigl \{ \sum _{i = 1}^n p_i^{(\delta )} \delta _{i - 1} + p^{(\delta )}_{n + 1} {{\,\mathrm{Blended}\,}}\Big ( \sum _{i = 1}^m p^{(e)}_i (\Gamma _{\alpha _i, \theta } + n - \tfrac{1}{2}), {{\,\mathrm{GPD}\,}}_{\mu = \kappa , \sigma , \xi = \xi }; p^{(b)}, \kappa , \varepsilon \Big ) \\& \Bigm \vert p^{(\delta )} \in [0, 1]^{n + 1}, p^{(e)} \in [0, 1]^m, p^{(b)} \in [0, 1]^2, \\& \sum p^{(\delta )}_i = \sum p^{(e)}_i = p_1^{(b)} + p_2^{(b)} = 1, \theta \in \mathbb {R}_+, \sigma \in \mathbb {R}_+ \Bigr \}. \end{aligned}$$

This family leads to $n_{\text {tail}} = n + m + 5$ and the concrete definition $f_{\text {adaptor}}(x) = (\theta , \sigma , p^{(\delta )}, p^{(e)}, p^{(b)}) =( \mathrm {sp}(x_{1}), \mathrm {sp}(x_{2}), \mathrm {sm}_{\mathbb {R}^{n+1}}(x_{3:n+3}),$ $\mathrm {sm}_{\mathbb {R}^m}(x_{n+4:n+m+3}), \mathrm {sm}_{\mathbb {R}^2}(x_{n+m+4:n+m+5}))'$ where $x_{i:j} = (x_i, x_{i+1}, \ldots , x_j)'$ denotes vector slices and where $\mathrm {sp}={{\,\mathrm{softplus}\,}}$ and $\mathrm {sm}={{\,\mathrm{softmax}\,}}$.

## Fitting the reporting delay model to truncated data

In this section, we describe a conditional maximum-likelihood-based approach for fitting Model 3 in detail. We will start by deriving the conditional likelihood function for observed reporting delays from Observation Scheme 1 under the general setting of Model 1, see Sect. 3.1. We then proceed by considering the global model from Model 2, and describe an estimation approach based on a modified EM-Algorithm, see Sect. 3.2. Once we have an estimate for the global parameters, we can use them as starting values for an estimation procedure for the micro-level model from Model 3, see Sect. 3.3. Not using good starting values for the micro-level model proved detrimental to convergence of the estimation routine to the point of becoming unusable.

### The conditional likelihood for truncated reporting delays

In this section we derive a conditional likelihood function for observed reporting delays from Observation Scheme 1 under the general setting of Model 1. It is worthwhile to mention that the resulting conditional likelihood is not bound to the case of reporting delays, but applies in any setting involving a parametric model for randomly truncated data, provided the model is dominated by a $\sigma$-finite measure and some (conditional) independence assumptions are met.

It follows from Model 1 that the reporting delays are conditionally independent given the claim features as well as the accident time, i.e.,

$$\begin{aligned} (D_{j}^{(i)} \vert X^{(i)}=x^{(i)}, T_{\mathrm {acc},j}^{(i)} = t, Y_{j}^{(i)} = y) \text { are independent with distribution } P_D(x^{(i)},t,y), \end{aligned}$$

for some distribution $P_D(x^{(i)},t,y)$ depending only on $x^{(i)},t, y$. While Models 2 and 3 are based on specific parametric assumptions, it is instructive to keep things universal, and only make the assumption that $P_D(x^{(i)},t,y)$ has cumulative distribution function $F_{g(x^{(i)},t,y)} \in \{F_{g(x^{(i)},t,y)}: g \in \mathcal {G}\}$ for some suitable family $\mathcal {F}= \{F_\theta : \theta \in \Theta \}$ of distributions that is dominated by some $\sigma$-finite measure $\mu$ (the $\mu$-densities are denoted by $f_\theta$), and for some family $\mathcal {G}$ of functions $g:\mathfrak {X}\times (0,\infty ) \times \mathfrak {Y}\rightarrow \Theta$ (in a global model, $\mathcal {G}$ would be the class of all constant functions $g \equiv \theta$ with $\theta \in \Theta$). Note that a natural dominating measure for the ${{\,\mathrm{BDEGP}\,}}$ family is $\mu = \text {Leb} + \sum _{i = 0}^{n - 1} \delta _i$.

To see how the data $\mathfrak {D}_\tau$ observed by an insurer at calendar time $\tau$ can be described as a truncated sample, consider points from $\xi = \xi ^{(i)}$ (for the sake of readability, we omit the upper index *i* for the moment). They contain $(t_{\text {acc},j}, d_{\mathrm {report},j})$, and are observed by the insurer if $t_{\text {acc},j} + d_{\mathrm {report},j} \le \tau$. Hence, every observed reporting delay is truncated to the interval $d_{\mathrm {report},j} \in [0, \tau - t_{\text {acc},j}]$. As a consequence, the likelihood of every observed reporting delay must be calculated conditional on the event $D_{j} \in [0, \tau - T_{\text {acc},j}]$, i.e.,

$$\begin{aligned} f_{D \vert D \in [0, \tau - T_{\mathrm {acc}}], X=x, T_{\text {acc}} = t_{\text {acc},j}, Y=y_{j} }(d_{j})&= \frac{f_{g(x, t_{\text {acc},j},y_{j})}(d_j)}{F_{g(x, t_{\text {acc},j},y_{j})} (\tau - t_{\text {acc},j})}, \end{aligned}$$

where $(x,t_{\mathrm {acc},j},y_j,d_j)=(x^{(i)},t_{\mathrm {acc},j}^{(i)},y_j^{(i)},d_j^{(i)})$. This leads to the following conditional log-likelihoods for Models 2 and 3, respectively:

8 $$\begin{aligned} \ell ^G(\theta \vert \mathfrak {D}_\tau )&= \sum _{(x,t, y,d) \in \mathfrak {D}_\tau } \log f_{\theta }(d) - \log F_{\theta }(\tau - t), \end{aligned}$$

9 $$\begin{aligned} \ell ^M(g \vert \mathfrak {D}_\tau )&= \sum _{(x,t, y,d) \in \mathfrak {D}_\tau } \log f_{g(x, t, y)}(d) - \log F_{g(x, t, y)}(\tau - t). \end{aligned}$$

Strategies to efficiently calculate the maximum of these functions are presented in the next two sections.

### Estimating the global model

In this section, we describe how to maximize $\theta \mapsto \ell ^G(\theta \vert \mathfrak {D}_\tau )$ from (8). In view of the fact that the underlying BDEGP family is essentially a mixture family, a natural approach consists of using a suitable version of the EM algorithm [14]. In fact, the procedure for fitting a BDEGP family to data is divided into subproblems which maximize conditional likelihoods on subsets of the parameter space. These building blocks need slight adaptations for blended distributions and Erlang mixture distributions, but are largely similar.

Before describing the algorithms, it is instructive to consider the underlying basics of a generic version of the EM algorithm that may be applied to samples of (both upper and lower) randomly truncated observations from a mixture model. Here, the generic mixture model shall be defined in terms of given parametric families $\mathcal F_1, \dots , \mathcal F_k$, where the *j*th component family $\mathcal F_j$ has $\mu$-density $f_{j,\theta _j}$ with parameter $\theta _j \in \Theta _j$, for some common dominating sigma-finite measure $\mu$ (often the sum of the Lebesgue measure on $\mathbb {R}$ and the counting measure on some subset of $\mathbb Z$). The mixture model, denoted $\mathcal F$, is then given by the family of $\mu$-densities that are of the form

10 $$\begin{aligned} f_{(p, \theta )}(x)&= \sum _{j = 1}^k p_j f_{j; \theta _j}(x) \end{aligned}$$

for some mixture weights $p \in (0, 1)^k$ (with $\sum _{j=1}^{k} p_j = 1)$ and some $\theta =(\theta _1, \dots , \theta _k) \in \Theta = \bigotimes _{j=1}^k \Theta _j$.

The fact that observations are truncated can be modelled as follows: let (*X*, *L*, *U*) denote a random vector, where *X* is the variable of interest that is supposed to have a mixture density $f_{(p,\theta )}$ as in (10). The pair (*L*, *U*) is assumed to be independent of *X* and shall satisfy $L \le U$, with *L* possibly equal to $-\infty$ and *U* possibly equal to $+\infty$. Further, (*L*, *U*) shall have a density $f_{(L,U)}$ with respect to some dominating sigma-finite measure $\nu$. A sample of interval truncated observations from (*X*, *L*, *U*) consists of independent observations $(x_i,\ell _i,u_i)$ that we only happen to see if $\ell _i \le x_i \le u_i$. As a consequence, any observed value can be regarded as being drawn from the $(\mu \otimes \nu )$-density

11 $$\begin{aligned} f_{(X,L,U) \mid L \le X \le U}(x,\ell ,u)&= \frac{f_{(L,U)}(\ell ,u) f_{(p,\theta )}(x)}{\Pr (L \le X \le U)} \varvec{1}(\ell \le x \le u). \end{aligned}$$

Subsequently, we write $(X^t, L^t, U^t)$ for a random vector following the above density, i.e,

$$\begin{aligned} f_{(X^t, L^t, U^t)}(x,\ell , u)=f_{(X,L,U) \mid L \le X \le U}(x,\ell ,u). \end{aligned}$$

Estimating $(p,\theta )$ based on plain maximum likelihood requires specifying a distribution for (*L*, *U*) (which can be regarded as a nuisance parameter) and calculating the denominator in (11). This (major) nuisance can be avoided by instead considering conditional maximum likelihood [3], which is known to produce consistent estimators as well. In our case, we rely on considering the density of $X^t$ conditional on the value of $(L^t,U^t)=(\ell , u)$, which is given by

$$\begin{aligned} f_{X^t \mid L^t=\ell , U^t=u}(x)&= \frac{f_{(X^t, L^t, U^t)}(x, \ell , u)}{f_{(L^t, U^t)}(\ell , u)} \\&= \frac{f_{(X,L,U) \mid L \le X \le U}(x,\ell ,u) }{\int _{[\ell ,u]} f_{(X,L,U) \mid L \le X \le U}(z,\ell ,u) \mathrm {\,d}z} = \frac{f_{(p,\theta )}(x)}{F_{(p,\theta )}([\ell , u])} \end{aligned}$$

for $\ell \le x \le u$, where $F_{(p,\theta )}([\ell , u])=\int _{[\ell ,u]} f_{(p, \theta )}(z) \mathrm {\,d}\mu (z)$. As can be seen, the conditional density/likelihood is independent of the distribution of (*L*, *U*), and hence easily accessible.

For later purposes, it is helpful to attach a weight $w_i$ to each observation $(\ell _i, x_i, u_i)$ (one might think of $w_i=1$ for the moment). Denote the resulting sample by $\mathfrak {I}= \mathfrak {I}_w = \{ (x_i, \ell _i, u_i, w_i) \vert \ell _i \le x_i \le u_i\},$ with sample size $N = |\mathfrak {I}|$. Based on the motivation in the previous paragraph, we aim at maximizing

12 $$\begin{aligned} \ell (p,\theta \vert \mathfrak {I}) = \sum _{(x, \ell , u, w) \in \mathfrak {I}} w \cdot \Big [ \log f_{(p,\theta )}(x) - \log F_{(p,\theta )}([\ell ,u]) \Big ], \end{aligned}$$

which is akin to maximizing $\ell ^G(\theta \vert \mathfrak {D}_\tau )$ from (8), after identifying $\ell =0$, $x=d_{\mathrm {report}}$, $u=\tau -t_{\mathrm {acc}}$ and $w=1$. An approximate maximizer of (12), say $(\hat{p}, \hat{\theta })$, may be obtained by Algorithm 1. 
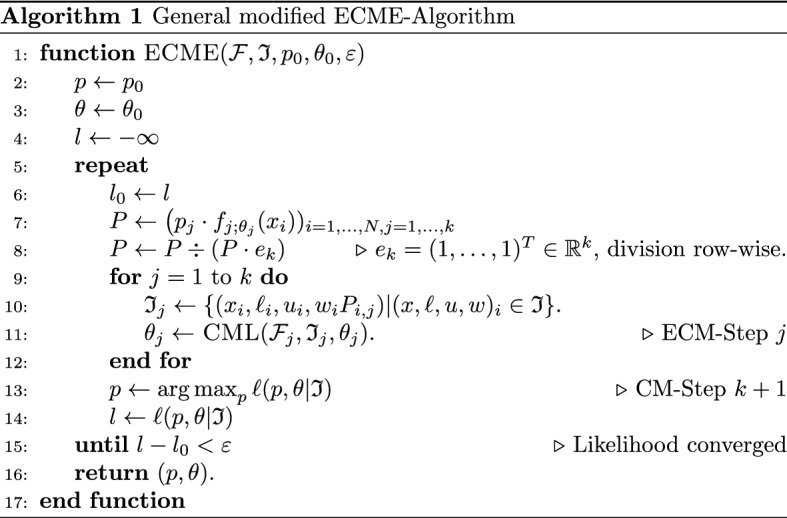


The algorithm can be motivated by the ECME principle (see [14, 27, 29]), and is derived in great detail in the supplementary material. The function $\textsc {CML}$ used in line 11 of Algorithm 1 is defined as follows: for some given family $\mathcal {H}$ consisting of densities $h_\theta$ parametrized by $\theta \in \Theta$ and given a truncated and weighted sample $\mathfrak {I}$, possibly utilizing a starting value $\theta _0 \in \Theta$ for assessing the following maximum numerically, let

13 $$\begin{aligned} \mathop {\text {\textsc {CML}}} (\mathcal {H}, \mathfrak {I}, \theta _0) :={{\,\mathrm{arg\,max}\,}}_{\theta \in \Theta } \sum _{(x, \ell , u, w) \in \mathfrak {I}} w \big [ \log h_\theta (x) - \log H_\theta ([\ell , u]) \big ], \end{aligned}$$

where $H_\theta$ is the corresponding distribution. Note that calculating the ${{\,\mathrm{arg\,max}\,}}$ can itself be based on applying an instance of an ECME algorithm if $\mathcal {H}$ is a mixture family (which is the case when applying Algorithm 1 to the BDEGP family from Definition 2). Furthermore, the densities $f_{j; \theta _j}$ used for computing the posterior probability matrix *P* in line 7 need to be with respect to the dominating $\sigma$-finite measure of $\mathcal {F}$, which may differ from the natural dominating measure of $\mathcal {F}_j$. This essentially leads to a separate treatment of discrete and continuous components since for each $x_i$ for which there exists a component *j* such that $\{x_i\}$ has positive probability over $\mathcal {F}_j$, $P_{i, j}$ will be zero for all components with zero probability of $\{x_i\}$ even if $x_i$ is in their support and has positive (Lebesgue) density.

Adaptations for blended distributions. In view of the fact that a blended distribution family (Definition 1) is of mixture type with $k=2$, we could in principle directly use the general ECME algorithm to calculate a maximizer of the associated weighted conditional log-likelihood. However, this would require working with transformed versions of the original blended families, see (5) and (6). Alternatively, in each ECM-step, one may optimise the weighted conditional log-likelihood with respect to the original families by transforming the data $\mathfrak {I}$ to the scale of the original families. More precisely, consider the first ECM step: if $\mathcal {F}_1 = \{f_{1;\theta _1}:\theta _1\in \Theta _1\}$ denotes the first component of the blended family ${{\,\mathrm{Blended}\,}}(\mathcal {F}_1, \mathcal {F}_2; \kappa , \varepsilon )$, then, in view of (5), the contribution of an observation $(x, \ell , u, w) \in \mathfrak {I}_1 \cap \{(x, \ell , u, w): x < \kappa + \varepsilon \}$ to the objective function is

$$\begin{aligned} \log f'_{1;\theta _1}(x) - \log F'_{1;\theta _1}([\ell , u])&= \log \frac{f_{1;\theta _1}(p(x)) \cdot \frac{\mathrm {\,d}}{\mathrm {\,d}\,x} p(x)}{F_{1;\theta _1}(\kappa )} - \log \frac{F_{1;\theta _1}([p(\ell ), p(u)])}{F_\theta (\kappa )} \\&= \log f_{1;\theta _1}(p(x)) + \log \tfrac{\mathrm {\,d}}{\mathrm {\,d}\,x} p(x) - \log F_{1;\theta _1}([p(\ell ), p(u)]), \end{aligned}$$

where $p = p_{\kappa , \varepsilon }$. Hence, instead of calculating $\textsc {CML}(\mathcal {F}'_1, \mathfrak {I}_1, \theta _1)$ (line 11 of Algorithm 1) we may equivalently calculate $\textsc {CML}(\mathcal {F}_1, \tilde{\mathfrak {I}}_1, \theta _1)$, where $\tilde{\mathfrak {I}}_1 :=\{ (p(x), p(\ell ), p(u), w) \mid (x, \ell , u, w) \in \mathfrak {I}_1 \}$ is the transformed dataset. An analogous result can be obtained for the second component $\mathcal {F}_2$, where the transformation uses $q=q_{\kappa , \varepsilon }$. Note that the associated transformed sample $\tilde{\mathfrak {I}}_2$ is a left-truncated sample, truncated at $\ell = \kappa - \varepsilon$. Overall, we obtain Algorithm 2, where we define $b_1(x) :=p_{\kappa , \varepsilon }(x)$ and $b_2 (x) :=q_{\kappa , \varepsilon }(x)$ for notational convenience. 
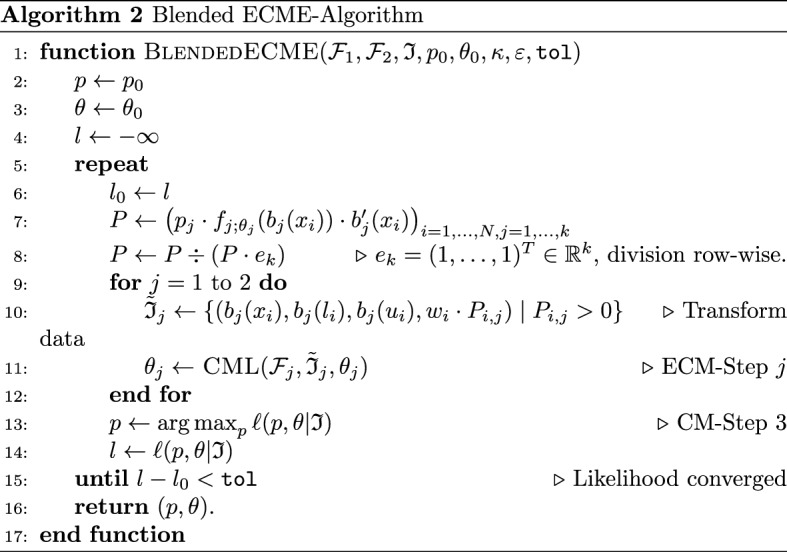


Adaptations for Erlang Mixtures. Erlang Mixture families $\mathcal {F}=\{\sum _{i = 1}^{k} p_i \cdot \Gamma _{\alpha _i, \theta }: p \in (0, 1)^k, \Vert p\Vert _1 = 1, \theta \in (0,\infty ),\alpha \in \mathbb {N}^k, \alpha _1< \ldots < \alpha _k \}$ do not satisfy the definition of a mixture-type family because they possess the additional constraint that each of the Erlang components has the same scale parameter. This prevents fitting Erlang mixtures based on direct applications of the EM or the ECME algorithm, see also [19, 25, 35] for related problems with no or constant truncation bounds. For our current setting of truncation bounds that may vary with each observation, we need to adapt ideas from these papers. In particular, we rely on a version of the ECME algorithm when treating the (integer) shape parameters as fixed, and then propose a shape search algorithm to solve the remaining integer optimization problem. Details are provided in Section C in the supplementary material, where we also explain the choice of starting values.

### Estimating neural networks

In this section we describe our approach to fitting a neural network model $\mathcal G$ as described in Sect. 2.3 based on maximization of (9) under Model 3 with the ${{{\,\mathrm{BDEGP}\,}}}_{\text {fix}}$ adaptation, i.e., holding $\alpha$ and $\xi$ fixed as obtained from fitting the global model. As a consequence, the loss function is $-\ell ^M(g \mid \mathfrak {D})$, which is a rather complex loss in comparison to standard losses used in ML. Training of the neural network was performed with the Adam algorithm [23] ($\text {lr} = 0.05, \beta _1 = \beta _2 = 0$) and stepsize reduction on plateau ($\text {factor} = 0.5, \text {patience} = 2, \text {min\_lr} = 10^{-4}, \text {min\_delta} = 10^{-6}$). TensorFlow [1] was used as the runtime for performing all necessary computations.

Once a specific network architecture $\mathcal G$ and a specific optimization routine have been chosen, fitting the neural network requires network initialization, i.e., choosing starting values for all free parameters of the model. The most common approach, introduced by Ref. [17], consists of global random initialization, where all free matrix parameters are i.i.d. uniform with mean zero and range dependent on the layer dimensions, and all free bias parameters are initialised to 0, which is then possibly applied repeatedly to potentially find better local optima. Such an approach, however, implies that the starting value of the distribution parameters feeding into the log-likelihood (9) is essentially random, and in view of the fact that the network is trained by direct optimization of the loss function, one may expect poor convergence (the latter has been confirmed in extensive preliminary experiments with simulated and real data). To alleviate this problem, we propose to only randomly initialise the free parameters of the embedding and hidden layers according to [17], while the output layer weights are chosen in such a way that the network output *g*(*x*, *t*, *y*), for each observation (*x*, *t*, *y*), is close to some pre-specified value, for instance the global estimate $\hat{\theta }$ from Sect. 3.2. This may be achieved by choosing the weights *A* and the bias *b* in the output layer in such a way that $A \approx 0$ has sufficiently small entries (see below) and $b= f^{-1}_{\text {adaptor}}(\hat{\theta })$, where $f^{-1}_{\text {adaptor}}: \Theta \rightarrow \mathbb {R}^{n_{\text {tail}}}$ is an inverse of the output layer link function $f_{\text {adaptor}}$ defined in Sect. 2.3.

We have experimented with three different approaches to initialise *A*: either $A \equiv 0$ (then $g(x, t, y) \equiv \hat{\theta }$ deterministically), or *A* initialised by the default initialization [17], or *A* initialised with small random parameters on the same scale as *b*, i.e. $A_{i, j} \sim U[-0.1, 0.1] \cdot b_i$ for all rows $i = 1, \ldots , n_{\text {tail}}$ and columns $j = 1, \ldots , n_{N_{\text {dense}}}$ where $n_{N_{\text {dense}}}$ is the dimension of the final hidden layer in the MLP architecture. The latter method, called scaled uniform initialization, proved most efficient in practice, see Sect. 5.2 for more details. The method is summarized in Algorithm S.1 in the supplementary material.

## Claims count prediction

The objective of claims reserving is to obtain good (aggregate) predictions for characteristics that depend on the partly unobserved paths of $\xi ^{(i)}$ across different time and feature sections, based on reported observations $\xi ^{(i)}_r$ as in Observation Scheme 1. Among such characteristics is for instance the afore-mentioned total number of IBNR claims at calendar time $\tau$, i.e.,

$$\begin{aligned} \sum _{i=1}^{N_{\text {pol}}} \sum _{j=1}^{N^{(i)}} \varvec{1}(T_{\mathrm {acc},j}^{(i)} \le \tau , T_{\mathrm {acc},j}^{(i)} + D_{\mathrm {report},j}^{(i)} > \tau ) = \sum _{i = 1}^{N_{\text {pol}}} \xi ^{(i)}(S_{\tau } ), \end{aligned}$$

where $S_{\tau } :=\{(t,y,d): t \le \tau , t+d> \tau \}$. A further object, of primal interest for insurance pricing, is given by the total number of claims in a given time period $[t_0, t_1]$, for instance an occurence year that is not necessarily related in any specific way to $\tau$, and for a certain class of risk feature $\mathfrak {X}' \subset \mathfrak {X}$ and claim features $\mathfrak {Y}' \subseteq \mathfrak {Y}$, i.e.,

$$\begin{aligned} \sum _{i:x^{(i)} \in \mathfrak {X}'} \sum _{j=1}^{N^{(i)}} \varvec{1}(T_{\mathrm {acc},j}^{(i)} \in [t_0,t_1], Y_j^{(i)} \in \mathfrak {Y}') = \sum _{i:x^{(i)} \in \mathfrak {X}'} \xi ^{(i)}([t_0,t_1] \times \mathfrak {Y}' \times [0, \infty )). \end{aligned}$$

The specific problems in the previous paragraph are special cases of the following task: for sets of interest $\mathfrak {X}'\subset \mathfrak {X}$ and $S = \{(t,y,d): t \in [t_0, t_1], y \in \mathfrak {Y}', d \in I_t\}$ with $[t_0, t_1] \subset [0,\infty )$, some $\mathfrak {Y}' \subset \mathfrak {Y}$ and some $I_t \subset [0,\infty )$, predict the unobserved individual (and/or aggregated) claim counts in *S*, i.e.,

$$\begin{aligned} N^{(i)}(S) = \xi ^{(i)}(S) \quad \text { and/or }\quad N(\mathfrak {X}' \times S) = \sum _{i:x^{(i)} \in \mathfrak {X}'}\xi ^{(i)}(S) \end{aligned}$$

based on a sample $\mathfrak {D}$ as in Observation Scheme 1. We will next discuss how such predictors may be derived based on knowledge of $P_D$ only, given some suitable homogeneity constraints are met. In practice, $P_D$ may be replaced by some estimate $\hat{P}_D$, for instance the neural network estimator from Sect. 3.3.

### Predictions under local homogeneity assumptions

We start by simplifying the prediction problem by restricting attention to predictors that depend on $\mathfrak {D}=\mathfrak {D}_\tau$ through the reported numbers $N^{(i)}_r(S)=\xi ^{(i)}_r(S)$ only. Let $\xi ^{(i)}_{nr}=\xi ^{(i)}-\xi ^{(i)}_r =\xi ^{(i)}(\,\cdot \, \cap R_\tau ^c)$ denote the claim arrivals that are *not reported* by calendar time $\tau$. By the restriction theorem (Theorem 5.2 in [24]), $\xi ^{(i)}_{nr}$ and $\xi ^{(i)}_{r}$ are independent Poisson processes with intensity measures $\mu ^{(i)}(\,\cdot \, \cap R_\tau ^c)$ and $\mu ^{(i)}(\,\cdot \, \cap R_\tau )$, respectively. As a consequence, the best $L^2$-predictor for $N^{(i)}(S)$ in terms of $N^{(i)}_r(S)$ is given by

14 $$\begin{aligned} \hat{N}^{(i)}(S) = \mathbb {E}[N^{(i)}(S) \mid N^{(i)}_r(S) ] = N^{(i)}_r(S) + \mathbb {E}[N^{(i)}_{nr}(S) ], \end{aligned}$$

and it remains to calculate the unconditional expectation on the right-hand side. Since

15 $$\begin{aligned} \mathbb {E}[N^{(i)}_{nr}(S) ] = \mu ^{(i)}(S \cap R_\tau ^c) = \int _{S \cap C^{(i)} \cap R_\tau ^c} \lambda (x^{(i)}, t) P_D (x^{(i)}, t,y)(\!\mathrm {\,d}d) P_Y(x^{(i)},t)(\!\mathrm {\,d}y) \mathrm {\,d}t \end{aligned}$$

by Campbell’s theorem, the latter boils down to calculating a complicated high-dimensional integral. The fact that calculation of this integral must be feasible in practice is a major demand when designing models for the distributions involved in Model 1, i.e., for $\lambda , P_Y$ and $P_D$. Under the following local homogeneity assumption, the calculation simplifies significantly.

#### Assumption 1

(Local homogeneity of claims developement) For a given interval $T \subset [0,\infty )$ and $\mathfrak {Y}'\subset \mathfrak {Y}$:

- $t \mapsto \lambda (x, t)=:\lambda (x)>0$ is constant on *T* for any *x*.
- $t \mapsto P_Y(x, t)(\mathfrak {Y}') =:P_Y(x)(\mathfrak {Y}')>0$ is constant on *T* for any *x*.
- $(t,y) \mapsto P_D(x,t,y)=:P_D(x)$ is constant on $T \times \mathfrak {Y}'$ for any *x*.

Even if the global claims process is highly inhomogeneous, these assumptions are approximately met for sufficiently small intervals *T* and sufficiently similar sets of claims $\mathfrak {Y}'$ (for continuity reasons, this particularly applies for the distributions $P_D$ from Model 3). For instance, [4] implicitly imposes Assumption 1 for all subsequent intervals of length corresponding to one month and for $\mathfrak {Y}'$ representing either material or injury claims. Under Assumption 1, we can simplify

$$\begin{aligned} \mu ^{(i)}(S)&= \int _{T \cap C^{(i)}} \int _{\mathfrak {Y}'} \int _{I_t} \lambda (x^{(i)}, t) P_D(x^{(i)}, t,y)(\!\mathrm {\,d}d) P_Y(x^{(i)}, t)(\!\mathrm {\,d}y) \mathrm {\,d}t \\&= \lambda (x^{(i)}) P_Y(x^{(i)})(\mathfrak {Y}') \int _{T \cap C^{(i)}} P_D(x^{(i)})(I_t) \mathrm {\,d}t, \end{aligned}$$

and likewise

16 $$\begin{aligned} \mu ^{(i)}(S \cap R_\tau )&= \lambda (x^{(i)}) P_Y(x^{(i)})(\mathfrak {Y}') \int _{T \cap C^{(i)}} P_D(x^{(i)})(I_t \cap [0, (\tau - t)_+]) \mathrm {\,d}t, \end{aligned}$$

17 $$\begin{aligned} \mu ^{(i)}(S \cap R_\tau ^c)&= \lambda (x^{(i)}) P_Y(x^{(i)})(\mathfrak {Y}') \int _{T \cap C^{(i)}} P_D(x^{(i)})(I_t \cap ((\tau - t)_+, \infty )) \mathrm {\,d}t. \end{aligned}$$

As a consequence of (17), the predictor in (14) greatly simplifies, with only univariate integrals to be calculated for each *i*. Moreover, the previous equations may be manipulated in such a way that one obtains a natural estimator for the expectation on the right-hand side of (14) that does not depend on $\lambda (x^{(i)})$ or $P_Y(x^{(i)})(\mathfrak {Y}')$. Indeed, by Eqs. (16) and (17),

$$\begin{aligned} \mu ^{(i)}(S \cap R_\tau ^c) = \mu ^{(i)}(S \cap R_\tau ) \cdot \frac{ \int _{T \cap C^{(i)}} P_D(x^{(i)})(I_t \cap ((\tau - t)_+, \infty )) \mathrm {\,d}t }{ \int _{T \cap C^{(i)}} P_D(x^{(i)})(I_t \cap [0, (\tau - t)_+]) \mathrm {\,d}t }, \end{aligned}$$

provided the denominator is positive. Replacing $\mu ^{(i)}(S \cap R_\tau )$ by its (unbiased) empirical analogue, $N_r^{(i)}(S)$, and then replacing the expectation on the right-hand side of (14) by the obtained expression, we finally obtain the predictor

18 $$\begin{aligned} \tilde{N}^{(i)}(S)&= N_r^{(i)}(S) \cdot \frac{ \int _{T \cap C^{(i)}} \hat{P}_D(x^{(i)})(I_t) \mathrm {\,d}t }{ \int _{T \cap C^{(i)}} \hat{P}_D(x^{(i)})(I_t \cap [0, (\tau - t)_+,]) \mathrm {\,d}t }, \end{aligned}$$

where $\hat{P}_D$ is a suitable estimate of $P_D$. Note that, for the important special case of $S = T \times \mathfrak {Y}' \times [0,\infty )$, i.e., $I_t = [0, \infty )$, the numerator further reduces to $\text {Leb}(T \cap C^{(i)})$.

Throughout the remaining parts of this paper, we will impose the homogeneity assumption from Assumption 1 for all intervals $((\ell - 1) \cdot p, \ell \cdot p]$ with $\ell = 1, \ldots , \lceil \tau / p\rceil$, and for all sufficiently small neighborhoods of points $y \in \mathfrak {Y}$. Note that the parameter *p* allows to control the restrictiveness of the local homogeneity assumption, which is less restrictive for smaller values of *p*. Given a set of features and accident times to be evaluated, say $A = \mathfrak {X}' \times [t_0, t_1] \times \mathfrak {Y}' \subset \mathfrak {X}\times [0,\tau ] \times \mathfrak {Y}$, we aim at predicting the number of claims in *A* that are reported within a given (calendar) time interval $(\tau _0, \tau _1]$ with $0 \le \tau _0< \tau _1\le \infty$, i.e.,

$$\begin{aligned} N_{\tau _0:\tau _1}(A) = \sum _{i: x^{(i)} \in \mathfrak {X}'} \xi ^{(i)}(S_{\tau _0:\tau _1}) \end{aligned}$$

with $S_{\tau _0: \tau _1} = \{(t, y, d): t \in [t_0, t_1], y \in \mathfrak {Y}', \tau _0 < t + d \le \tau _1\}$. Note that $N_{0:\infty }(A)$ corresponds to the *ultimate number of claims in* *A*, while $N_{\tau :\tau +q}(A)$, is the *number of claims in* *A* *that are reported within a period of length* $q>0$ *after calendar time* $\tau$. The argumentation that lead to (18) suggests the following predictor for $N_{\tau _0:\tau _1}(A)$ based on observed values $\mathfrak {D}_{\tau }$:

19 $$\begin{aligned} \hat{N}_{\tau _0:\tau _1}^{p}(A; \mathfrak {D}_{\tau })&:=\sum _{(x, t, y, d) \in (A \times \mathbb {R}_+) \cap \mathfrak {D}_{\tau }} \frac{\int _{I_p(x, t)\cap [t_0, t_1]} \hat{P}_D(x, t, y)((\tau _0 - s, \tau _1 - s]) \mathrm {\,d}s}{ \int _{I_p(x, t)\cap [t_0, t_1]} \hat{P}_D(x, t, y)([0, \tau - s]) \mathrm {\,d}s}, \end{aligned}$$

where $\hat{P}_D(x, t, y) \approx P_D(x, t, y)$ is the estimated reporting delay distribution and $I_p(x, t) = C(x) \cap (p \cdot \lfloor t/p \rfloor , p \cdot (\lfloor t / p \rfloor + 1)]$ with *C*(*x*) the coverage period of policy *x* and $(p \cdot \lfloor t/p \rfloor , p \cdot (\lfloor t / p \rfloor + 1)]$ the interval containing accident time *t* on which $\xi ^{(i)}$ is assumed homogeneous.

Note that the predictor in (19) can be updated continuously with the passing of time, either by reestimating $\hat{P}_D$ and then recalculating the predictor (which is computationally expensive), or by just updating the predictor with the estimated model $\hat{P}_D$ held fixed/updated only once in a while (which is less expensive). The main computational cost for predictor updates with fixed $\hat{P}_D$ lies in evaluation of the univariate integral in (19).

### Evaluating claim count predictors

For comparing different methods we define evaluation metrics that measure prediction errors in a standardized way. These evaluation metrics will be used in case studies to compare model performance, as well as to perform model selection in a backtesting context. All methods will be supplied with a sample $\mathfrak {D}_{\tau }$ as in Observation Scheme 1.

A generic predictor for $N_{\tau _0:\tau _1}(A)$ based on observations $\mathfrak {D}_{\tau }$ is denoted by $\hat{N}_{\tau _0:\tau _1}(A; \mathfrak {D}_{\tau })$. For simplicity, we only consider $(\tau _0, \tau _1)=(0,\infty )$ and $(\tau _0, \tau _1)=(\tau , \tau +q)$, which correspond to the ultimate number of claims and to the number of claims reported within the next period of length $q \in \{365, 365 / 4, 365 / 12\}$ (measured in days), respectively. For evaluating the predictor, we restrict attention to sets

$$\begin{aligned} A_{q, \ell , \mathfrak {Y}'} = \mathfrak {X}\times [(\ell - 1) \cdot q, \ell \cdot q) \times \mathfrak {Y}', \end{aligned}$$

where $\ell \in \{1, \dots , \tau / q\}$ denotes the $\ell$th period and $\mathfrak {Y}' \subset \mathfrak {Y}$. Root-mean-squared-error performance measures are then used to evaluate the performance, i.e.,

20 $$\begin{aligned} \text {RMSE}_{\tau _0:\tau _1}(\mathfrak {Y}', q)&:=\bigg ( \frac{q}{\tau } \cdot \sum _{\ell = 1}^{\tau / q} \big ( \hat{N}_{\tau _0:\tau _1}(A_{q, \ell , \mathfrak {Y}'}; \mathfrak {D}_{\tau }) - N_{\tau _0:\tau _1}(A_{q, \ell , \mathfrak {Y}'}) \big )^2 \bigg )^{\frac{1}{2}}, \end{aligned}$$

considered for $(\tau _0, \tau _1)=(0,\infty )$ and for $(\tau _0, \tau _1)=(\tau , \tau +q)$. Note that other error measures were examined as well, but the subsequent presentation is restricted to the above choices. In a real world scenario, $\text {RMSE}_{\tau _0:\tau _1}$ is computable from $\mathfrak {D}_{\tau _1}$ at calendar time $\tau _1$, enabling use of error measures with $\tau _1 < \infty$ outside of laboratory settings where the ground truth is known.

## Simulation study

To demonstrate the effectiveness of the micro-level approach compared to a classical Chain Ladder based approach, we compare predictors arising from the two methods on simulated data from Model 1. Apart from a homogeneous portfolio with constant exposure, we also examine how the methods perform in the presence of smooth or abrupt changes in the claim arrival process.

### Simulating car insurance portfolios

The portfolios considered throughout the simulation study build upon the car insurance data set described in Appendix A in [36]. The latter data set provides claim counts for 500,000 insurance policies, where each policy is associated with the risk features

$$(\mathtt {age}, \mathtt {ac}, \mathtt {power}, \mathtt {gas}, \mathtt {brand}, \mathtt {area}, \mathtt {dens}, \mathtt {ct}),$$

which correspond to age of driver, age of car, power of car, fuel type of car, brand of car, and area code, respectively; see also (A.1) in [36] for further details. Next to that, the data set also provides the variable truefreq, which corresponds to the claim intensity $\lambda (x)$ in our model. Note that the precise functional relationship $x \mapsto \lambda (x)$ has not been published by the authors.

In the following, we describe how the above data set was used to define nine different portfolios meeting the model assumptions described in Model 1 (in particular, we need to introduce a dynamic component, claim features as well as reporting delays). Each portfolio is considered over ten periods of 365 days, that is, the portfolio coverage period is the interval [0, 3650]. We start with a baseline setting that corresponds to the classical *homogeneous portfolio*.

#### Scenario 1: a homogeneous portfolio

The homogeneous portfolio is characterized by a homogeneous *exposure* as well as position-independent *claim intensity*, *occurrence process*, and *reporting process*. It may be considered the vanilla portfolio that practitioners often aim at by careful selection of considered risks and suitable transformations, e.g., adjustment for inflation.

Exposure. New risks arrive according to a homogeneous Poisson process with intensity $50,000/365 \approx 137$ and contracts are assumed to run for exactly one year (the latter could be extended to some non-trivial annual churn rate; however, the fact that some of the considered claim features depend on calendar time and we do not know the true functional from of $x\mapsto \lambda (x)$ prevent us from doing this). Moreover, the portfolio starts with exactly 50,000 policies with $t_{\text {start}} = 0$ and with remaining contract duration that is uniform on [0, 365]. As a consequence, the total exposure is constant in expectation and we have $N_{\text {pol}} \sim {50,000} + {{\,\mathrm{Poi}\,}}({500,000})$. Finally, for each risk in the portfolio we randomly draw (with replacement) risk features from the aforementioned data set from [36].

Claim Intensity. The claim frequency $\lambda (t, x) = \lambda (x)$ is independent of *t* and $t_{\text {start}}$ and given by the variable truefreq that belongs to the risk selected in the previous paragraph.

Occurrence Process. The occurrence process is position-independent, i.e., $P_Y(x, t) = P_Y(x)$. In view of the fact that the original data set from [36] does not contain any individual claim variables, we employed a simple but realistic process that fits into the setting of motor liability claims. More precisely, we choose to work with two claim variables, $y = (\mathtt {cc}, \mathtt {severity})$, with claims code $\mathtt {cc} \in \{ \text {injury}, \text {material} \}$, and claim size $\mathtt {severity} \in \mathbb {R}_+$. The claim feature distribution of cc is chosen to be a function of the policy features ac, power, and dens in such a way that material damages are more likely to occur in densely populated areas and with low-powered and newer cars (see Appendix D in the supplement for details on the precise relationship). The claim severity distribution of severity is log-normal with $\sigma$ constant and with $\mu$ depending on cc, brand, ac and power in such a way that injury claims, especially with older high-powered cars, are more severe. Moreover, material damages for certain premium brands are also more severe. Again, details are provided in Appendix D in the supplement.

Reporting Process. The reporting process is position-independent, i.e, $P_D(x, t, y) = P_D(x, y)$. We choose to work with $P_D(x, y) \in {{\,\mathrm{BDEGP}\,}}(n = 1, m = 3, \kappa = 3 \cdot 365, \varepsilon = 365 / 2)$ as a basic family, with fixed erlang shapes $\alpha = (1, 3, 6)$ that do not depend on *x* and *y*. The remaining 7 parameters (i.e., the four mixture weights of $\delta _0, \Gamma (1, \theta ), \Gamma (3, \theta ), \Gamma (6, \theta )$, and ${{\,\mathrm{GPD}\,}}(\kappa , \sigma , \xi )$, as well as $\theta , \sigma$, and $\xi$) are chosen to depend on age, dens, ac (only if cc is material), cc, and severity in such a way that more severe claims, material claims with new cars, and claims with younger drivers in populated areas will be reported sooner, while low-severity injuries will be reported later; see Appendix D in the supplement for details.

**Fig. 3:**
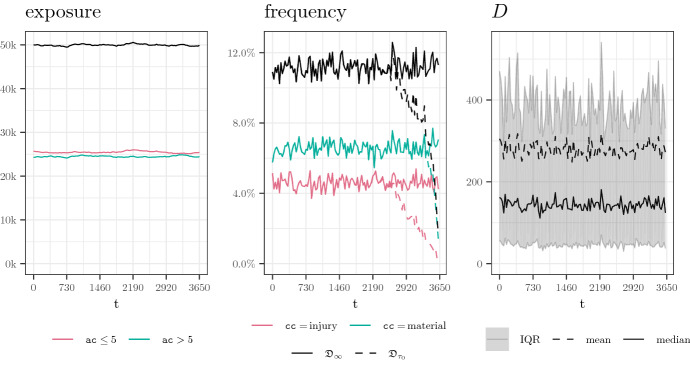
A path simulated from the baseline scenario. Left: exposure by time *t* (i.e. active policies; $\# \{ t \in C^{(i)} \vert i \in \{1, \ldots , N_{\text {pol}} \}$) Center: claims frequency by accident time *t* (i.e. mean number of claims per policy and year). Reported claims (dashed) and occurred claims (solid). Right: monthly summary statistics of reporting delay *D* by accident time *t*

A simulated portfolio from the baseline setting is illustrated in Fig. 3.

#### Scenarios 2a and 2b: changes in the exposure

The baseline setting from Scenario 1 is modified in such a way that the exposure is not constant, but either changes smoothly or abruptly in time.

In practice, smooth changes may result from a shift in the risk class distribution within the portfolio, for instance due to the fact that a competitor introduces a new product which is more attractive than the insurers own product for some risk class. In such a case, adverse selection would cause a shift in the newly written risks as the competitor product gains more visibility in the market. On the other hand, abrupt changes in the exposure may be caused by the introduction of a new risk class within the portfolio, for instance as a consequence of the introduction of a completely new product to the market, or the addition of a new sales channel reaching a new target group. Likewise, abrupt removal of an existing risk class may occur if underwriting policies change such that a product is no longer sold to a certain group, or if external factors such as OEM-provided insurance make the product obsolete for some risks.

For the simulation study, a smooth shift in exposure is realized by gradually reducing the proportion of new cars insured ($\mathtt {ac} \le 5$); see Appendix D in the supplement for details. Over the course of the simulation, the expected proportion of contracts with new cars reduces gradually from the starting value of 51.15–$9.48\%$. An abrupt change is introduced in the same way, by abruptly lowering the expected proportion of new cars insured to $9.48\%$ halfway through the simulation.

#### Scenarios 3a and 3b: changes in the claim intensity

The baseline setting from Scenario 1 is modified in such a way that the claim intensity is not constant, but either changes smoothly or abruptly in time.

In practice, smooth shifts in the claim intensity may result from improved security devices reducing the risk of accidents by prevention. Preventive measures could also be implemented by the insurer, e.g. by rewarding safer driving styles in insurance telematics products [7]. On the other hand, abrupt changes may be caused by the introduction of a product with extended coverage or by external factors such as reduced traffic volume and thus decreased risk of traffic accidents, for instance due to COVID-19 related lockdown measures.

For the simulation study, a smooth shift of the claim intensity is realized by reducing the individual claim frequencies by $20\%$ over the course of the simulation. Note that this also implies non-uniform occurrences. A shock is introduced by abruptly lowering the individual claim frequencies by 20% halfway through the simulation.

#### Scenarios 4a and 4b: changes in the occurrence process

The baseline setting from Scenario 1 is modified in such a way that the occurrence process is not constant, but either changes smoothly or abruptly in time.

In practice, smooth shifts in the claim feature distribution can be caused by a gradual macroeconomic or social change such as developments on the labor market. Abrupt changes in the claim feature distribution can be caused by external factors such as highly publicized events covered by the insurance in question. A practical example for legal insurance would be the Volkswagen emissions scandal 2015.

For the simulation study, a shift in the occurrence process is realized by making the probability $P(\mathtt {cc} = \text {material})$ depend on the accident time. More precisely, the probability is chosen to increase from 58.73 to $77.51\%$ ($+0.9$ on a logit scale for each risk). In addition, the severity distribution for material claims gets an increase by 1 in log-$\mu$ whereas injuries have a decrease of 0.5 in log-$\mu$ and an increase of 0.5 in log-$\sigma$. A shock is introduced in the same way, by abruptly increasing the probability of material claims and the severity distributions halfway through the simulation.

#### Scenarios 5a and 5b: changes in the reporting process

The baseline setting from Scenario 1 is modified in such a way that the reporting process is not constant, but either changes smoothly or abruptly in time.

In practice, smooth shifts in the reporting delay distribution could be caused by adaption of a new optional method for reporting claims, such as a customer portal. An abrupt change in the reporting delay distribution could be caused by introducing a new product with specific requirements for the claims reporting process, or by a legislative change in the definition of accident occurrence.

For the simulation study, a shift in the reporting process is realized by gradually increasing the probabilities $p_0$ and $p_1$ of the $\delta _0$ and $\Gamma (1, \theta )$ components by 2 on the logit scale, linearly with the accident time. The $\Gamma (3, \theta )$ component is also shifted such that the equation $p_2 = (1 - p_1) \cdot p_1$ still holds, see Appendix D in the supplement for details. A shock is introduced in the same way, by abruptly changing these probabilities halfway through the simulation.

**Fig. 4:**
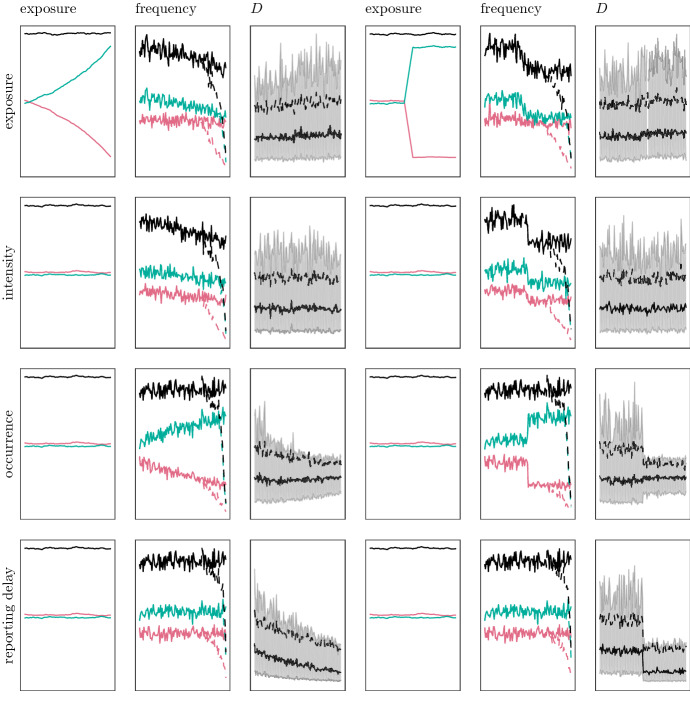
Overview of all drift (left column) and shock (right column) scenarios applied to the various components (rows). The plots are explained in Fig. 3

Simulated portfolios from the eight non-homogeneous scenarios are illustrated in Fig. 4. Note that Scenarios 2a–3b do not yield large changes in the monthly summary statistics of the reporting delays, which suggests that IBNR prediction in these scenarios is simpler than in Scenarios 4a–5b.

### Details on neural network predictors

Recall the generic predictor from (19), which depends on an estimated reporting delay distribution $\hat{P}_D(x,t,y)$ and a homogeneity parameter *p*. When employing the neural network estimator from Sect. 3.3, we denote the resulting predictor by $\hat{N}^{\text {NNet}, p}_{\tau _0:\tau _1}$.

For computational reasons, we choose the correct BDEGP model specification (i.e., the ${{\,\mathrm{BDEGP}\,}}(n = 1, m = 3, \kappa = 3 \cdot 365, \varepsilon = 365 / 2)$ family with unknown parameters) throughout the simulation study, as additional model selection is not feasible within a large scale simulation experiment. Note however that model selection was successfully applied in the real data application in Sect. 6.

A number of further choices have to be made for modelling and estimating $P_D$, the most crucial ones concerning the neural network architecture, the activation function and the weight initialization. In view of the fact that a case-by-case choice based on training and validation sets is computationally too demanding for a large-scale simulation study, we chose to fix one particular choice based on the results of a preliminary experiment in the baseline setting. The results are presented in Table 1; they concern the $\text {RMSE}_{0:\infty }(\mathfrak {Y}, 365)$-performance measure each evaluated in 5000 simulation runs (50 portfolios with 100 initial weights), and suggest to use the $\mathrm {softplus}$ activation function, the scaled uniform initialization strategy and the neural network architecture with $N_{\text {dense}} = 1$ hidden layer of size $n_1 = 5$.

**Table 1:** Median from 5000 runs of $\text {RMSE}_{0:\infty }(\mathfrak {Y}, 365)$ for different hyperparameter choices, after 2000 epochs in the baseline setting. The Adam optimizer was used with 2000 epochs and parameters hand tuned to $\mathrm {lr} = 0.05$, $\beta _1 = \beta _2 = 0$. Hyperparameters are tuned one-by-one, the other parameters being held at $\mathrm {softplus}$, $N_{\text {dense}} = 1$, $n_1 = 5$, and scaled uniform initialization

Activation function		Architecture		Weight initialisation	
	$\text {RMSE}_u$		$\text {RMSE}_u$		$\text {RMSE}_u$
Relu	69.638	5	68.398	$A \leftarrow 0$	68.414
Softplus	68.398	10	69.382	$A_i \leftarrow U[-0.1, 0.1]^{\otimes n_{N_{\text {dense}}}} \cdot b_i$	68.398
		10,5	71.600	$A \leftarrow$ [17]	69.377
		15,10,5	73.687		

Next, in view of the well-known nuisance that neural network training crucially depends on the initial network weights, a procedure is needed to choose among fits calculated from various initial weights. A natural approach consists of choosing the fit with the smallest loss. However, extensive experiments not shown in detail for the sake of brevity suggest that the following approach, partly tailored to the prediction problem at hand, yields substantially better results: among all candidate predictors (we use 100 initial weights for each data set), keep the one which minimizes the yearly backtesting error

$$\begin{aligned} \widehat{\text {RMSE}}_{0:\infty }(\hat{N}_{0:\infty }; \mathfrak {Y}, 365) :=\bigg ( \frac{1}{9} \cdot \sum _{\ell = 1}^{9} \big ( \hat{N}_{0:\infty }(A_{365, \ell , \mathfrak {Y}}; \mathfrak {D}_{\tau - 365}) - \hat{N}_{0:\infty }(A_{365, \ell , \mathfrak {Y}}; \mathfrak {D}_{\tau }) \big )^2 \bigg )^{\frac{1}{2}}, \end{aligned}$$

which is obtained from $\text {RMSE}_{0:\infty }(\mathfrak {Y}, 365)$ in Eq. (20) by plugging in $\hat{N}_{0:\infty }(\dots ; \mathfrak {D}_{\tau })$ for $N_{0:\infty }(\dots )$ and evaluating on $\hat{N}_{0:\infty }(\dots ; \mathfrak {D}_{\tau - 365})$. Note that the selection does not involve any data unseen by time $\tau$.

### Details on Chain Ladder predictors

Similar to the NNet predictor, the Chain Ladder method was used with different discretization periods $p\in \{365, 365 / 4, 365 / 12\}$. Based on cumulative link ratios $f_{j}=f_j(p)$ for development period $j \in \{1, \dots , \tau / p - 1\},$

21 $$\begin{aligned} f_j&= \frac{ \# \{ (x, t, y, d) \in \mathfrak {D}_{\tau } : \lfloor \tfrac{t + d}{p} \rfloor \le \lfloor t / p \rfloor + j \le \tau / p \} }{ \# \{ (x, t, y, d) \in \mathfrak {D}_{\tau } : \lfloor \tfrac{t + d}{p} \rfloor +1 \le \lfloor t / p \rfloor + j \le \tau / p \} }, \end{aligned}$$

the Chain Ladder predictors, for $A \subset \mathfrak {X}\times \mathbb {R}_+ \times \mathfrak {Y}$, are given by

$$\begin{aligned} \hat{N}^{\text {CL}, p}_{0:\infty } (A)&= \sum _{i = 1}^{3650 / p} \hat{N}^{\text {CL}, p}_{0:\infty }(A \cap A_{p,i, \mathfrak {Y}}) \\&= \sum _{i = 1}^{3650 / p} N_r(A \cap A_{p,i,\mathfrak {Y}}) \cdot \prod _{j = \tau / p - i + 1}^{\tau / p - 1} f_j \\ \hat{N}^{\text {CL}, p}_{\tau :\tau +365} (A)&= \sum _{i = 1}^{3650 / p} \hat{N}^{\text {CL}, p}_{\tau :\tau +365}(A \cap A_{p,i,\mathfrak {Y}}) \\&= \sum _{i = 1}^{3650 / p} N_r(A \cap A_{p,i,\mathfrak {Y}}) \cdot \Biggl ( \prod _{j = \tau / p - i + 1}^{(\tau + 365) / p - i \wedge \tau / p - 1} f_j - 1 \Biggr ). \end{aligned}$$

Note that, unlike the neural network predictors, the Chain Ladder method can only be updated in discrete time steps which are multiples of the discretization period.

In view of the fact that cc is the main feature causing the perturbations in the non-homogeneous scenarios, we also applied Chain Ladder separately for $\text {cc} \in \{\text {material}, \text {injury}\}$, resulting in the predictors

$$\begin{aligned} \hat{N}^{\text {CL}_{cc}, p}_{0:\infty }(A)&= \sum _{i = 1}^{3650 / p} \sum _{c \in \{\text {material}, \text {injury}\}} N_r(A \cap A_{p,i, \{c\} \times \mathbb {R}_+}) \cdot \prod _{j = \tau / p - i + 1}^{\tau / p - 1} f^c_j, \\ \hat{N}^{\text {CL}_{cc}, p}_{\tau :\tau + 365}(A)&= \sum _{i = 1}^{3650 / p} \sum _{c \in \{\text {material}, \text {injury}\}} N_r(A \cap A_{p,i, \{c\} \times \mathbb {R}_+}) \cdot \Biggl ( \prod _{j = \tau / p - i + 1}^{(\tau + 365) / p - i \wedge \tau / p - 1} f^c_j - 1 \Biggr ), \end{aligned}$$

where the Chain Ladder factors $\{f^c_j\}_j$ are calculated as in (21), but with $\mathfrak {D}_{\tau }$ replaced by $\mathfrak {D}_{\tau } \cap \{ \mathtt {cc}=c\}$.

### Results

Throughout this section we highlight important findings from the simulation study.

**Fig. 5:**
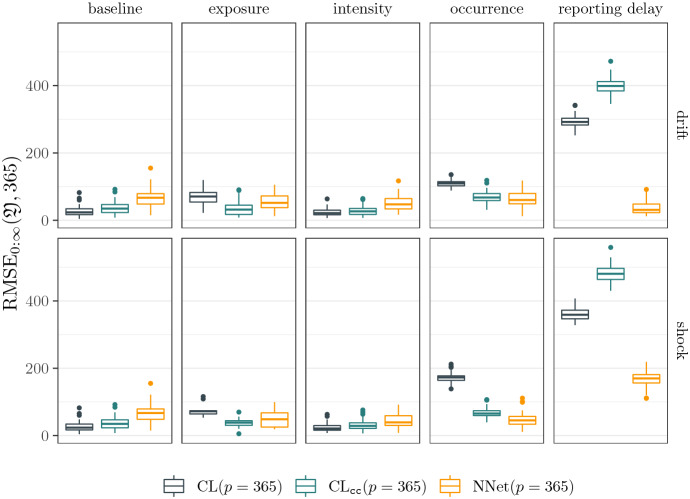
Boxplots of the overall error measure $\text {RMSE}_{0:\infty }(\mathfrak {Y}, 365)$, each based on $n = 50$ simulated paths

We start by providing a general overview of the performance across scenarios. For the sake of illustration, we restrict attention to three predictors only, namely $\hat{N}^{\text {CL}, 365}_{0:\infty }, \hat{N}^{\text {CL}_{cc}, 365}_{0:\infty }$ and $\hat{N}_{0:\infty }^{\text {NNet},365}$, and to the evaluation metric $\text {RMSE}_{0:\infty }(\mathfrak {Y}, q)$ with $q = 365$ (other predictors and evaluation periods lengths *q* will be considered below). The results are summarized in Fig. 5, where we depict, for each scenario described in Sect. 5.1, boxplots of the evaluation metric obtained from 50 simulation runs each. We observe that, for the baseline setting as well as for Scenarios 3a and 3b (Intensity), both Chain Ladder methods exhibit a slightly smaller overall error than the neural network predictor. This behavior may have been expected, since the global reporting delay distribution and thus the development pattern which Chain Ladder relies on is essentially constant over time in the two scenarios, as can be seen from Figs. 3 and 4. Within Scenarios 2a and 2b (Exposure), the global Chain Ladder predictor performs slightly worse that the neural network predictor, while $\mathrm {CL}_{cc}$ performs best. The latter may be explained by the fact that the introduced inhomogeneities have rather little influence on the frequency of the injury claims (see Fig. 4), whence restricted Chain Ladder performs well on that subset. The neural network predictor shines in Scenarios 4 and 5 (Occurrence and Reporting Delay, respectively), which both exhibit rather large inhomogeneities in the reporting process (see Fig. 4). These two scenarios greatly deteriorate the performance of Chain Ladder, while the neural network is able to adapt to the changes. Summarizing the findings, we find that the neural network predictor works reasonably well in all situations under consideration, with rather minor disadvantages in some scenarios, and substantial advantages in others.

**Fig. 6:**
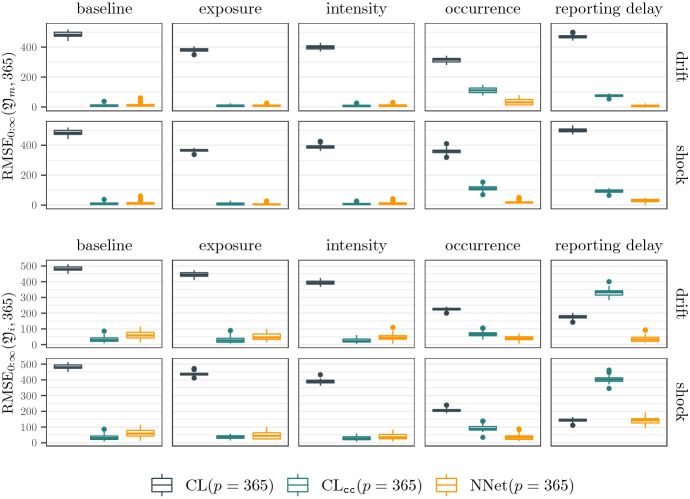
Boxplots of error measures split by claims code $\text {RMSE}_{0:\infty }(\mathfrak {Y}_m, 365)$ and $\text {RMSE}_{0:\infty }(\mathfrak {Y}_i, 365)$, each based on $n = 50$ simulated paths

Next, in Fig. 6, we report analogous results separately by claims code for the performance measures $\text {RMSE}_{0:\infty }(\mathfrak {Y}_m, 365)$ and $\text {RMSE}_{0:\infty }(\mathfrak {Y}_i, 365)$, where $\mathfrak {Y}_m :=\{\text {material}\} \times \mathbb {R}_+$ and $\mathfrak {Y}_i :=\{\text {injury}\} \times \mathbb {R}_+$ are the subsets of $\mathfrak {Y}$ restricted to a single claims code. The message is simple: for all scenarios under consideration, the plain Chain Ladder predictor is unable to provide accurate, competitive predictions on the subsets defined by $\mathtt {cc} = \text {material}$ and $\mathtt {cc} = \text {injury}$. When comparing the neural network predictor with $\mathrm {CL}_{cc}$, we observe the same qualitative behavior as in Fig. 5. It is, however, important to mention that the latter method requires prior identification of the relevant features (which might not be possible), while the neural network approach is universal, and can be applied with ease to any evaluation set of interest.

**Fig. 7:**
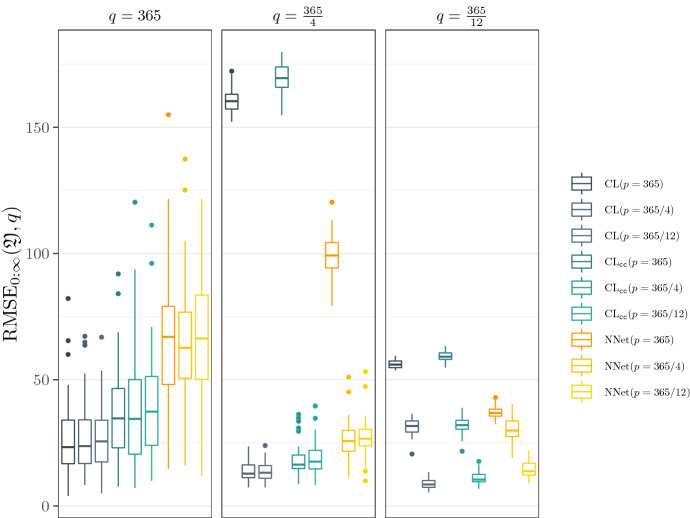
Boxplots of error measure $\text {RMSE}_{0:\infty }(\mathfrak {Y}, q)$ for different evaluation periods $q \in \{ 365, 365/4, 365/12 \}$ in the baseline setting

Finally, the results presented in Fig. 7 allow to compare the predictors with development period $p \in \{365, 365/4, 365/12 \}$ with respect to performance measures with evaluation period $q \in \{365, 365/4, 365/12\}$. For the sake of brevity, we restrict attention to the baseline setting; qualitatively similar results were obtained for the other scenarios. We observe that, if the development period is larger than the evaluation period, the errors tend to increase drastically, in particular for the Chain Ladder method. On the other hand, if the development period is smaller than the evaluation period, the error increases slightly showing reduced stability. Overall it seems preferable to choose the smallest development period that still yields stable results as the period of choice. Another observation that can be made is that the difference between Chain Ladder and neural network based approaches gets smaller for shorter evaluation periods. In other words, the stability advantage of Chain Ladder with its comparatively few parameters diminishes as the number of Chain Ladder parameters (link ratios to be estimated) increases—even in the optimal setting for chain ladder where the portfolio and the occurrence process is homogeneous.

## Application to real data

Throughout this section, we compare our new approach with Chain Ladder in an application to a large real dataset containing motor legal insurance claims provided by a German insurance company. Details on the dataset are provided in Sect. 6.1. The methods and results, including strategies applied for model selection (which have been omitted in Sect. 5), are discussed in Sect. 6.2. In a nutshell, the results show that the neural network predictors robustly provide more efficient predictions than Chain Ladder.

### The dataset

The dataset contains a portfolio of about 250,000 motor legal insurance contracts with exposure information available monthly from 31st January 2014 to 31st December 2020. Information on the claims of this portfolio is available up to 31st December 2020. as well. In total, there are about 65,000 reported claims.

The policy features considered for modelling reporting delays are

$$(\mathtt {tstart}, \mathtt {cstart}, \mathtt {tariff}, \mathtt {dob}),$$

where

- tstart is the start date of the contract.
- cstart is the start date of the customer relationship.
- tariff gives information on the tariff (regular, public service, self-employed).
- dob contains the date of birth of the customer (accurate to months, contains missing values). Missing values were imputed using the median observed age at contract start (tstart) as a reference. An indicator variable to show missingness was also added.

In addition, several low-cardinality claim features available at time of reporting, as well as the accident time were included:

$$(\mathtt {tacc}, \mathtt {cc}, \mathtt {covered}, \mathtt {channel}, \mathtt {reporter}),$$

where

- tacc is the accident date. The dataset contains inaccurate data, where the true accident time is unknown. These are encoded as January 1st and flagged with an indicator variable. Moreover, some rare claims have $\mathtt {tacc} = \mathtt {tstart}$ (which, for instance, is due to legal consulting regarding claims that have happened before the contract has started); these claims are identified with an additional indicator variable.
- cc is the claims code, a rough categorization of the type of claim. It has five different categories numbered from 1 to 5.
- covered is the coverage status of the claim. It has four different categories, but is almost binary (covered, not covered, partially covered, coverage status unknown), with ‘covered’ and ‘not covered’ making up the majority of cases.
- channel is the channel by which the claim was reported. It has six different categories (mail, e-mail, fax, online, in person, telephone).
- reporter denotes the reporter of the claim. It has six different categories, but is almost binary (policyholder, additional insured, lawyer, intermediary, other, unknown). Most claims are reported by the policyholder or filed directly by a lawyer.

The rationale for including tacc as a feature in the neural network predictors is to help identify drifts in the reporting process.

Due to the extreme shock the COVID-19 pandemic had on the dataset, we chose to only consider data available up to 31st December 2019 for model evaluation, since none of the prediction methods provided remotely acceptable results when validating the predicted number of claims given data up to 31st December 2019 compared to the actual numbers observed in 2020.

**Fig. 8:**
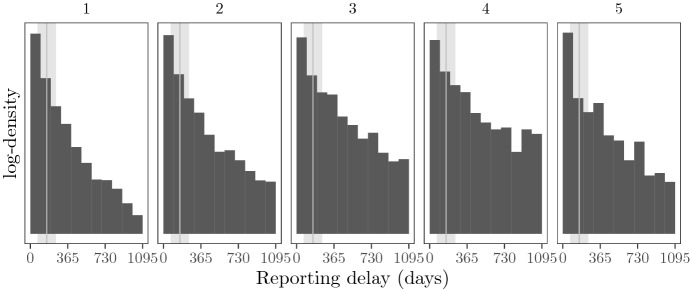
Empirical reporting delay distributions by cc. Logarithmic axis. Vertical lines mark the chosen blending region $\kappa$ and $\kappa \pm \varepsilon$

### Results

Predictions were calculated based on a conventional Chain Ladder approach as well as on various neural network predictors (among which a final, data-adaptive choice was made as described below). To reduce the effect of a single calendar year on our studies, we examined two artificial truncation points, $\tau = \text {31st December 2017}$ and $\tau = \text {31st December 2018}$, and evaluated the methods using the one-year-ahead validation error $\mathrm {RMSE}_{\tau :\tau +365}(\mathfrak {Y}, q = 365)$ for both truncation points.

Regarding Chain Ladder, we chose to separately apply it to the five datasets defined by the different claims code (which corresponds to $\mathrm {CL}_{cc}$ from the previous section, and could be regarded as common actuarial practice). A visualization of the different reporting delay distributions by cc can be found in Fig. 8. Note that further subdivision may severely impact the stability of Chain Ladder methods, due to the combinatorial explosion of the number of different link ratio sequences that would have to be estimated.

Regarding the neural network predictors, the underlying ${{\,\mathrm{BDEGP}\,}}$ family was specified as follows: first, we held $\kappa = 160$ and $\varepsilon = 90$ fixed; mainly for computational reasons. Next, we chose to consider all combinations of $n \in \{7, 14, 21, 30\}$ and $m \in \{3, 5, 10, 15\}$. After computing global fits for all these families, we proceeded to repeatedly train a neural network model with fixed architecture $n_1 = 10, n_2 = 5$ for 2000 epochs, each ten times with different random starting values. During training, the available data were split into a training and a validation set in a ratio of $75\%: 25\%$. The loss, i.e., the mean negative log-likelihood, was monitored for both datasets and logged for each epoch. This process was repeated for both data truncation points $\tau$.

**Fig. 9:**
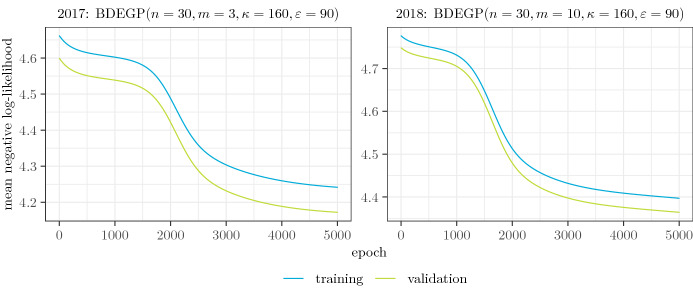
Training and validation losses by epoch for the final selected models per $\tau$

In an effort to reduce computational cost, only the six best families according to the mean backtesting error $\mathrm {RMSE}_{\tau -365:\tau }(\mathfrak {Y}, q = 365)$ over both years were trained for additional 3000 epochs with the same method. The selected families correspond to $(n, m) \in \{ (30, 10), (30, 3), (7, 10), (7, 15), (7, 3), (7, 5) \}$. The training and validation loss as a function of the epochs is exemplarily illustrated in Fig. 9 for the final selected models $(n,m)=(30,3)$ with $\tau = \text {31st December 2017}$ and $(n,m) = (30,10)$ with $\tau = \text {31st December 2018}$, which shows big loss improvements after 2000 epochs and a final loss close to convergence after 5000 epochs. Remarkably, the training loss is larger than the validation loss, which is due to a fortunate selection of the validation set.

Next, in order to select a final unique model, we performed model selection as follows: first, the best model per family (i.e, the best of ten random seeds for parameter initialization) was selected based on validation loss. Next, the overall model among the six remaining candidates was selected based on the backtesting error $\mathrm {RMSE}_{\tau -365:\tau }(\mathfrak {Y}, q = 365)$. Note that the latter evaluates the trained model on data it has already partially seen during training, which is readily available, and that it is a selection criterion which, unlike the final log-likelihood, allows for comparison across different architectures.

**Fig. 10:**
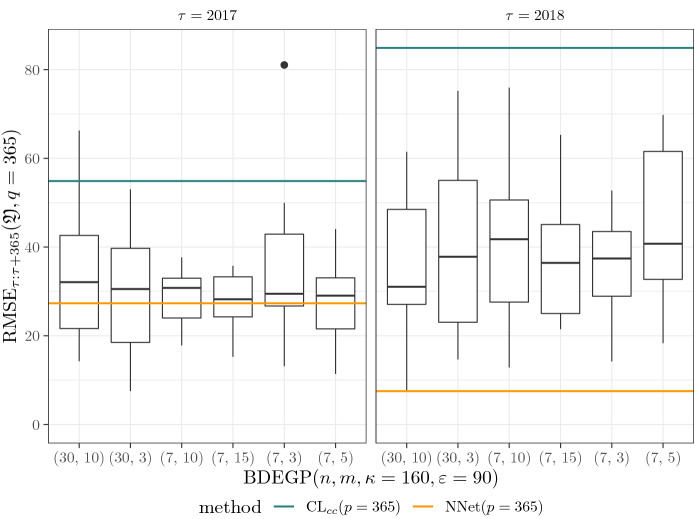
Comparison of $\text {RMSE}_{\tau :\tau +365}(\mathfrak {Y}, q = 365)$ for all runs with 5000 epochs. Horizontal lines show the corresponding values for $\text {CL}_{cc}$ and the final selected model

The results for the six models that were trained 5000 epochs, as well as the final selection and the Chain Ladder benchmark, are summarized in Fig. 10, where the 10 runs per model and year ($\tau$) are illustrated by a boxplot. Horizontal lines show the corresponding values for Chain Ladder and for the final selected model.

It can be seen that, irrespective of the model or the random seed used for parameter initialization, the neural network predictors outperform Chain Ladder, with very few exceptions for 2017 and huge improvements for most cases. In view of the fact that the final selected models show a substantially different performance for 2017 and 2018, the model selection procedure should be taken with some care. Nevertheless, even a random choice would provide a viable selection criterion, which shows that the neural network approach is quite robust with respect to model selection.

**Fig. 11:**
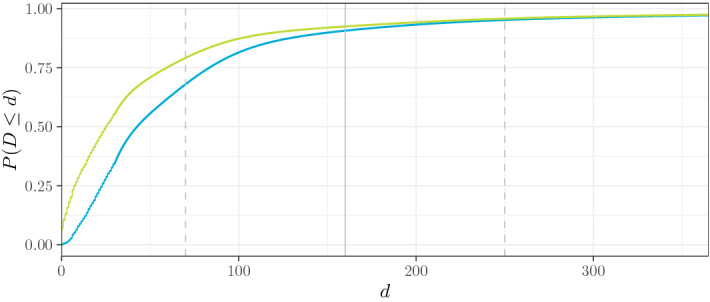
Predicted reporting delay distributions for two claims from the final fit with $\tau = \text {31st December 2017}$. Note the discreteness of the distributions on $0 \le d < 30$

Finally, predicted distributions for two exemplary claims in the training set are shown in Fig. 11 for $\tau = \text {31st December 2017}$. The claims have claim codes 1 and 2 respectively and show the flexibility of the ${{\,\mathrm{BDEGP}\,}}(30, 3, 160, 90)$ family underlying the neural network model.

## Conclusion

A new, flexible micro-level model for reporting delays has been developed and applied to predict IBNR claim counts. It was demonstrated that the approach performs well in comparison to classical actuarial methods in both simulation studies and on real world data. Strengths of the micro-level approach particularly emerge in the presence of heterogeneity in the data generating process, as is often the case in real world examples, and in the presence of complex relationships involving many features. Incorporating many features into classical methods becomes prohibitively difficult with an ever-increasing amount of available information. Another advantage of the newly developed method is the ability to continuously update predictions as new data becomes available, reducing critical information delay for stakeholders.

There are ample opportunities for further development on the approach:

1. The BDEGP family, while very flexible, might not be suitable for all applications. Future work could examine the choice of different families.
2. While Model 3 assumes a neural network functional relationship between reporting delay distribution and predictors, different functional relationships could be examined. The non-trivial nature of the conditional likelihood under study makes finding alternative functional forms with corresponding estimation techniques an interesting task.
3. The chosen MLP architecture is rather simple. Other architectures could be examined for their performance for the problem at hand.
4. The proposed claim count predictors are based on the the number of reported claims. This leads to the prediction becoming identical to zero if, in a particular portfolio, no claims were yet observed. By developing methods for estimating $P_Y$ and $\lambda$, access to a predictor based on (15) would allow to overcome this disadvantage.

## Supplementary Information

Below is the link to the electronic supplementary material.

Supplementary file1 (PDF 359 KB)

Supplementary file2 (PDF 39 KB)

## Data Availability

The script used for simulation studies is provided as supplementary material. It requires an R package hosted on GitHub [34]. The real dataset used in Sect. 6 is proprietary.

## References

[1] Abadi M, Agarwal A, Barham P, Brevdo E, Chen Z, Citro C, Corrado GS, Davis A, Dean J, Devin M, Ghemawat S, Goodfellow I, Harp A, Irving G, Isard M, Jia Y, Jozefowicz R, Kaiser L, Kudlur M, Levenberg J, Mané D, Monga R, Moore S, Murray D, Olah C, Schuster M, Shlens J, Steiner B, Sutskever I, Talwar K, Tucker P, Vanhoucke V, Vasudevan V, Viégas F, Vinyals O, Warden P, Wattenberg M, Wicke M, Yu Y, and Zheng X (2015) TensorFlow: Large-scale machine learning on heterogeneous systems. https://www.tensorflow.org/. (**Software available from tensorflow.org**)

[2] Allaire J, Eddelbuettel D, Golding N and Tang Y (2016) tensorflow: R interface to tensorflow. https://github.com/rstudio/tensorflow

[3] Andersen EB (1970). Asymptotic properties of conditional maximum-likelihood estimators. J Roy Stat Soc.

[4] Antonio K, Plat R (2014). Micro-level stochastic loss reserving for general insurance. Scand Actuar J.

[5] Antonio K, Godecharle E and Van Oirbeek R (2016) A multi-state approach and flexible payment distributions for micro-level reserving in general insurance. 10.2139/ssrn.2777467

[6] Arjas E (1989). The claims reserving problem in non-life insurance: some structural ideas. ASTIN Bull.

[7] Arvidsson S (2010). Reducing asymmetric information with usage-based automobile insurance. Swedish Natl Road Transp Res Inst (VTI).

[8] Baudry M, Robert CY (2019). A machine learning approach for individual claims reserving in insurance. Appl Stoch Model Bus Ind.

[9] Chollet F, Allaire J, et al (2017) R interface to keras. https://github.com/rstudio/keras

[10] De Felice M, Moriconi F (2019). Claim watching and individual claims reserving using classification and regression trees. Risks.

[11] Delong Ł, Wüthrich MV (2020). Neural networks for the joint development of individual payments and claim incurred. Risks.

[12] Delong Ł, Lindholm M and Wüthrich MV (2021) Collective reserving using individual claims data. Scandinavian Actuarial J 1–28

[13] Delong Ł, Lindholm M, Wüthrich MV (2021). Gamma mixture density networks and their application to modelling insurance claim amounts. Insur Math Econ.

[14] Dempster AP, Laird NM, Rubin DB (1977). Maximum likelihood from incomplete data via the EM algorithm. J Roy Stat Soc.

[15] Dozat T (2016) Incorporating Nesterov momentum into Adam. https://openreview.net/pdf/OM0jvwB8jIp57ZJjtNEZ.pdf

[16] Embrechts P, Mikosch T, Klüppelberg C (1997). Modelling extremal events: for insurance and finance.

[17] Glorot X and Bengio Y (2010) Understanding the difficulty of training deep feedforward neural networks. In: Teh YW and Titterington M (eds) Proceedings of the thirteenth international conference on artificial intelligence and statistics, volume 9 of proceedings of machine learning research, pp 249–256, Chia Laguna Resort, Sardinia, Italy. PMLR. http://proceedings.mlr.press/v9/glorot10a.html

[18] Goodfellow IJ, Bengio Y and Courville A (2016) Deep learning. MIT Press, Cambridge. http://www.deeplearningbook.org

[19] Gui W, Rongtan H, Lin X (2018). Fitting the erlang mixture model to data via a Gem-CMM algorithm. J Comput Appl Math.

[20] Hastie T, Tibshirani R, Friedman J (2009). The elements of statistical learning.

[21] Holden L and Haug O (2009) A mixture model for unsupervised tail estimation. http://arxiv.org/abs/0902.4137

[22] Jin X (2013) Micro-level loss reserving models with applications in workers compensation insurance. https://sites.google.com/site/xiaolijin2013/research/working-paper2

[23] Kingma DP and Ba J (2015) Adam: a method for stochastic optimization. In: Proceedings of the third international conference on learning representations. ICLR’15. http://arxiv.org/abs/1412.6980

[24] Last G, Penrose M (2018). Lectures on the poisson process.

[25] Lee SCK, Lin XS (2010). Modeling and evaluating insurance losses via mixtures of erlang distributions. North Am Actuarial J.

[26] Lee SC, Lin XS (2012). Modeling dependent risks with multivariate erlang mixtures. ASTIN Bull.

[27] Liu C, Rubin DB (1994). The ECME algorithm: a simple extension of EM and ECM with faster monotone convergence. Biometrika.

[28] Lopez O, Milhaud X (2021). Individual reserving and nonparametric estimation of claim amounts subject to large reporting delays. Scand Actuar J.

[29] Meng X-L, Rubin DB (1993). Maximum likelihood estimation via the ECM algorithm: a general framework. Biometrika.

[30] Mikosch T (2009). Non-life insurance mathematics.

[31] Norberg R (1993). Prediction of outstanding liabilities in non-life insurance. ASTIN Bull.

[32] Norberg R (1999). Prediction of outstanding liabilities II. Model variations and extensions. ASTIN Bull.

[33] Radtke M, Schmidt KD, Schnaus A (2016). Handbook on loss reserving.

[34] Rosenstock A (2021) Reservr. https://github.com/AshesITR/reservr

[35] Verbelen R, Gong L, Antonio K, Badescu A, Lin S (2015). Fitting mixtures of erlangs to censored and truncated data using the EM algorithm. ASTIN Bull.

[36] Wüthrich MV and Buser C (2019) Data analytics for non-life insurance pricing. 10.2139/ssrn.2870308

[37] Wüthrich MV (2018). Neural networks applied to chain-ladder reserving. Eur Actuar J.

[38] Wüthrich M (2018). Machine learning in individual claims reserving. Scand Actuar J.

