# Supplementary material for: Micro-level prediction of outstanding claim counts based on novel mixture models and neural networks
Source: Eur Actuar J. 2022 May 12;13(1):55–90. doi: 10.1007/s13385-022-00314-4 (PMC9098157; doi:10.1007/s13385-022-00314-4)
Supplement: Supplementary file 1 — Supplementary file1 (PDF 359 KB) [file 13385_2022_314_MOESM1_ESM.pdf]

Supplementary Material: Micro-level Prediction of Outstanding Claim Counts using Neural Networks

Axel Bücher [1*] and Alexander Rosenstock [1,2]

[1*] Mathematisches Institut, Heinrich-Heine-Universität Düsseldorf, Universitätsstraße 1, 40225 Düsseldorf, Germany.
[2] ARAG SE, ARAG-Platz 1, 40472 Düsseldorf, Germany.

*Corresponding author(s). E-mail(s): axel.buecher@hhu.de;
Contributing authors: alexander.rosenstock@hhu.de;

Abstract

This supplement contains more details on the neural network model and its estimation (Appendix A), on the general ECME-algorithm (Appendix B) and its adaptations for fitting Erlang mixtures (Appendix C), and on the model specifications used in the simulation study (Appendix D). Throughout, arabic section numbers always refer to the main paper.

Appendix A Details on the neural network architecture

In this section we provide a detailed description of the neural network architecture used for modelling the reporting delay distribution in Section 2.3.

Basics on neural networks can be found in [1]. Each function $g \in \mathcal{G}$ shall be a composition of different layer functions, each with their own set of independent parameters. For a general overview of the structure of g, we refer to Figure A1. It aids understanding to give an informal summary of the structure of $\mathcal{G}$ before diving into the formal definition:

- The *head* of the network consists of embedding layers for categorical features and scaling transformations for continuous features, which serves

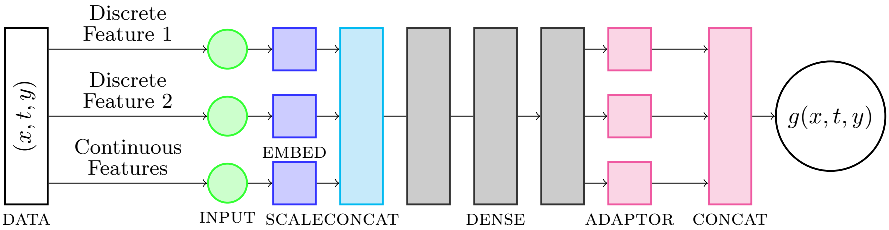

Figure A1: Schematic flow of input features to output for a neural network architecture with three hidden layers. The network will be trained with (9) as its loss.

to create a uniform-scale, continuous representation of all input features. The uniformity of scale is particularly important to conserve numerical stability in the presence of random initialization, see Section 3.3 for details.

- The *body* of the network aims at transforming the preprocessed features into an internal representation of the available information, based on one or more densely connected layers. This 'narrow' encoding of the data serves to extract the most important features with respect to the output variable (reporting delay in our case).
- The *tail* of the network aims at transforming the rich internal representation into concrete distributional parameters $\theta \in \Theta$. Intuitively, the tail doesn't need more complexity because all relevant structure is captured already in the internal representation.

We start by describing the *input layer*, which does not contain any free model parameters. Given an input $(x,t,y) \in \mathfrak{X} \times \mathbb{R} \times \mathfrak{Y}$, we first reorder the features, putting N_d discrete features to the front and N_c continuous features (e.g. t) to the back of a new intermediate vector. Furthermore, we shall code the ith discrete feature as an integer in $\{1,\ldots,c_i\}$ where $c_i \in \mathbb{N}$ is the number of different values that feature i can attain. Overall, we obtain the input layer function

$$g_{\text{in}} : \mathfrak{X} \times \mathbb{R} \times \mathfrak{Y} \to \prod_{i=1}^{N_d} \{1,\ldots,c_i\} \times \mathbb{R}^{N_c}$$

Next, for each of the discrete features, a so-called *embedding layer* is needed to transform the feature into a continuous variable for further use. For the ith discrete feature, the embedding function $g_{\text{embed},i} : \{1,\ldots,c_i\} \to \mathbb{R}^{d_i}$ has a fixed hyperparameter d_i, the *embedding dimension*, and $d_i \cdot c_i$ free parameters, denoted by $\alpha_1,\ldots,\alpha_{c_i}$ with $\alpha_j \in \mathbb{R}^{d_i}$, and is defined as

$$g_{\text{embed},i} : \{1,\ldots,c_i\} \to \mathbb{R}^{d_i}, \quad v \mapsto \alpha_v.$$

$g_{\text{embed},i}(v) = \alpha_v \in \mathbb{R}^{d_i}$ is called the *embedding of value v*. As illustrated in [2], continuous features should be brought to a common scale to improve

the numerical condition of the problem. This is realized with an affine transformation with parameters $\mu \in \mathbb{R}^{N_c}, \sigma \in \mathbb{R}_+^{N_c}$, namely $g_{\text{scale}} := x \mapsto \frac{x-\mu}{\sigma}$ with component-wise division to center and scale continuous features. When fitting the neural network (see Section 3.3), we employ empirical means and standard deviations, respectively. The overall *embedding layer* of the neural network is defined as

$$g_{\text{embed}} : \prod_{i=1}^{N_d} \{1, \ldots, c_i\} \times \mathbb{R}^{N_c} \to \mathbb{R}^{\sum_{i=1}^{N_d} d_i + N_c}, \quad g_{\text{embed}} := \bigotimes_{i=1}^{N_d} g_{\text{embed},i} \otimes g_{\text{scale}}$$

which is a function with a total of $\sum_{i=1}^{N_d} c_i \cdot d_i$ free parameters. We write

$$g_{\text{head}} := g_{\text{embed}} \circ g_{\text{in}} : \mathfrak{X} \times \mathbb{R} \times \mathfrak{Y} \to \mathbb{R}^{\sum_{i=1}^{N_d} d_i + N_c}.$$

After the head function that transforms all inputs into continuous variables, a series of *densely connected* layers (dense layers for short) are applied. The total number $N_{\text{dense}} \in \mathbb{N}$ of dense layers is to be considered as a hyperparameter of the model, and the same holds for the output dimension n_j, the *number of nodes*, of the jth layer. For brevity, we write $n_0 := N_c + \sum_{i=1}^{N_d} d_i$. The jth dense layer is then defined as

$$g_{\text{dense},j} : \mathbb{R}^{n_{j-1}} \to \mathbb{R}^{n_j}, \quad v \mapsto g_{\text{dense},j}(v) := \text{softplus}(Av + b),$$

where $A \in \mathbb{R}^{n_j \times n_{j-1}}$ and $b \in \mathbb{R}^{n_j}$ are the free parameters and where the nonlinear function softplus : $x \mapsto \log(1 + e^x)$, called the activation function, is applied coordinate-wise. The composition of the N_{dense} dense layers defines a function

$$g_{\text{body}} := g_{\text{dense},N_{\text{dense}}} \circ \cdots \circ g_{\text{dense},1} : \mathbb{R}^{\sum_{i=1}^{N_d} d_i + N_c} \to \mathbb{R}^{n_{N_{\text{dense}}}}$$

with $\sum_{j=1}^{N_{\text{dense}}} (n_{j-1} + 1) \cdot n_j$ free parameters. When fitting the model (see Section 3.3), we also experimented with $g_{\text{dense},j}(v) := \text{ReLU}(Av + b)$ but found results to be worse than with the smooth softplus activation function.

Composing g_{head} and g_{body} gives a function $g_{\text{body}} \circ g_{\text{head}} : \mathfrak{X} \times \mathbb{R} \times \mathfrak{Y} \to \mathbb{R}^{n_{N_{\text{dense}}}}$ which now needs to be mapped to the parameter set Θ of the chosen reporting delay distribution family $\mathcal{F}$ in order to obtain a usable family $\mathcal{G}$. A common feature of the families $\mathcal{F}$ that we invoke is that the parameter space Θ factors into (possibly bounded) intervals and probability parameters, that is parameters p constrained to $[0, 1]^d$ with $\|p\|_1 = 1$ (a method to cope with integer parameters present in Erlang mixtures and derived distributions is presented below). We can thus construct a natural *adapter family* of functions

$g_{\text{tail}} : \mathbb{R}^{n_{N_{\text{dense}}}} \to \Theta$ as follows: assume

$$\Theta = \mathbb{R}^{N_1} \times (0,\infty)^{N_2} \times (0,1)^{N_3} \times \prod_{i=1}^{N_P} [0,1]^{p_i} \cap S^1_{p_i},$$

where S^1_n denotes the unit sphere in $\mathbb{R}^n$ with respect to the 1-norm. Note that other interval constraints can be trivially reproduced by adding affine transformations, e.g. if $\theta_1 \in (a,b)$ is a constraint in the original parametrization, substitute $\tilde{\theta_1} = \frac{\theta_1 - a}{b-a} \in (0,1)$ as an equivalent parameter. The adaptor function for the $\mathrm{BDEGP}_{\text{fix}}(n,m,\kappa,\varepsilon,\alpha,\xi)$ family used in the paper is obtained by setting $N_1 = 0, N_2 = 2, N_3 = 0, N_P = 3, p_1 = n+1, p_2 = m, p_3 = 2$ in this general parametrization and ordering the free parameters $(\theta, \sigma, p^{(\delta)}, p^{(e)}, p^{(b)})$.

With $n_{\text{tail}} = n_{N_{\text{dense}}+1} := N_1 + N_2 + N_3 + \sum_{i=1}^{N_P} p_i$ the dimension of Θ, we define

$$\begin{aligned}
\text{sigmoid} &: \mathbb{R} \to (0,1), \quad x \mapsto \frac{\exp(x)}{\exp(x)+1} \\
\text{softmax}_{\mathbb{R}^n} &: \mathbb{R}^n \to (0,1)^n \cap S^1_n, \quad x \mapsto \Big(\exp(x_i) / \sum_{j=1}^n \exp(x_j) \Big)_{i=1}^n \\
f_{\text{adaptor}} &: \mathbb{R}^{n_{\text{tail}}} \to \Theta, \quad f_{\text{adaptor}} := \mathrm{Id}_{\mathbb{R}^{N_1}} \otimes \bigotimes_{i=1}^{N_2} \text{softplus} \otimes \bigotimes_{i=1}^{N_3} \text{sigmoid} \otimes \bigotimes_{i=1}^{N_P} \text{softmax}_{\mathbb{R}^{p_i}} \\
g_{\text{tail}} &: \mathbb{R}^{n_{N_{\text{dense}}}} \to \Theta, \quad v \mapsto f_{\text{adaptor}}(Av + b) \qquad \text{(S1)}
\end{aligned}$$

where $A \in \mathbb{R}^{n_{\text{tail}} \times n_{N_{\text{dense}}}}$ and $b \in \mathbb{R}^{n_{\text{tail}}}$ are free parameters. Finally, $\mathcal{G}$ is defined as the collection of all $g = g_{\text{tail}} \circ g_{\text{body}} \circ g_{\text{head}}$. $\mathcal{G}$ is a family with

$$n_\Psi = \sum_{i=1}^{N_d} c_i \cdot d_i + \sum_{j=1}^{N_{\text{dense}}} (n_{j-1}+1) n_j + (n_{\text{dense}}+1) n_{\text{tail}}$$

free real parameters. We denote the natural parametrization of $\mathcal{G}$ by $\Psi = \mathbb{R}^{n_\Psi}$ and identify a network $g \in \mathcal{G}$ by its n_Ψ weights $\psi \in \Psi$.

The output layer link function f_{adaptor} has a preimage given by

$$f^{-1}_{\text{adaptor}} = \mathrm{Id}_{\mathbb{R}^{N_1}} \otimes \bigotimes_{i=1}^{N_2} \text{softplus}^{-1} \otimes \bigotimes_{i=1}^{N_3} \text{sigmoid}^{-1} \otimes \bigotimes_{i=1}^{N_P} \text{softmax}^{-1}_{\mathbb{R}^{p_i}}$$

with $\text{softmax}^{-1}_{\mathbb{R}^{p_i}}(p) = \big(\log(p_j) - \log(\|p\|_\infty) \big)_{j=1}^{p_i}$ and $\|p\|_\infty$ the maximum norm of p (note that softplus and sigmoid are invertible). This is useful for choice of initial values based on a global estimate $\hat{\theta}$ during neural network initialization.

Algorithm S.1 Network Initialization

function InitialiseNNet($\mathcal{G}, \hat{\theta}$)
  $\psi \leftarrow ()$
  for $i = 1, \dots, N_d$ **do** ▷ Initialise embedding layers
    Draw $\alpha \sim U[-0.05, 0.05]^{d_i \otimes c_i}$
    Append α to ψ
  end for
  for $j = 1, \dots, N_{\text{dense}}$ **do** ▷ Initialise dense layers
    $l \leftarrow \sqrt{\frac{6}{n_j + n_{j-1}}}$
    Draw $A \sim U[-l, l]^{n_j \otimes n_{j-1}}$ ▷ [3]
    $b \leftarrow \vec{0} \in \mathbb{R}^{n_j}$
    Append (A, b) to ψ
  end for
  $b \leftarrow f^{-1}_{\text{adaptor}}(\hat{\theta})$ ▷ Initialize tail
  $A \leftarrow \text{Diag}(b) \cdot U[-0.1, 0.1]^{n_{\text{tail}} \otimes n_{N_{\text{dense}}}}$
  Append (A, b) to ψ
  Flatten $\psi \in \mathbb{R}^{n_\Psi}$
end function

Appendix B Derivation of the ECME Algorithm

In this section we provide a detailed motivation of Algorithm 1 given in Section 3.2. We follow the notation of the section in the paper.

First note that the conditional density of truncated observations is again of mixture type:

$$f_{X^t|L^t=\ell,U^t=u}(x) = \sum_{j=1}^{k} p_j \frac{f_{j;\theta_j}(x)}{F_{(p,\theta)}([\ell,u])} = \sum_{j=1}^{k} \tilde{p}_{j;p,\theta}(\ell,u) \tilde{f}_{j;\theta_j}(x;\ell,u), \qquad \ell \le x \le u, \tag{S2}$$

where, using the notation $F_{j;\theta_j}([\ell,u]) = \int_{[\ell,u]} f_{j;\theta_j}(z)\,\mathrm{d}\mu(z)$,

$$\tilde{p}_{j;p,\theta}(\ell,u) = p_j \cdot \frac{F_{j;\theta_j}([\ell,u])}{F_{(p,\theta)}([\ell,u])}, \qquad \tilde{f}_{j;\theta_j}(x;\ell,u) = \frac{f_{j;\theta_j}(x)}{F_{j;\theta_j}([\ell,u])}, \qquad \ell \le x \le u. \tag{S3}$$

The EM-algorithm for calculating a maximizer of $\ell(p,\theta|\mathfrak{I})$ is based on regarding the observations (ℓ_i, x_i, u_i) as being incomplete, in view of the fact we do not know from which conditional truncated component density $\tilde{f}_{j;\theta_j}(\,\cdot\,;\ell_i,u_i)$, see (S3), the observation $x_i \sim (X^t \mid (L^t,U^t) = (\ell_i,u_i))$ was simulated. More formally let $Z = Z(\ell,u) = (Z_1,\dots,Z_k)$, conditioned on $(L^t,U^t) = (\ell,u)$, follow a multinomial distribution $Z|(L^t = \ell, U^t = u) \sim \text{Mult}(1, \tilde{p}_{1;p,\theta}(\ell,u), \dots, \tilde{p}_{k;p,\theta}(\ell,u))$, i.e., $\Pr(Z = z|L^t = \ell, U^t = u) =$

$\tilde{p}_{1;p,\theta}^{z_1}(\ell, u) \cdot \ldots \cdot \tilde{p}_{k;p,\theta}^{z_k}(\ell, u)$ for $z = (z_1, \ldots, z_k) \in \{0, 1\}^k$ with $\sum_{j=1}^k z_j = 1$. If, conditional on $(L^t, U^t) = (\ell, u)$ and $Z_j = 1$, the random variable X^t has density $\tilde{f}_{j;\theta_j}$ from (S3), then the conditional distribution of X^t given $(L^t, U^t) = (\ell, u)$ is precisely given by (S2). In view of this representation, we now regard each (x_i, ℓ_i, u_i) as an incomplete observation of $(x_i, \ell_i, u_i; z_{i,1}, \ldots, z_{i,k})$ where $z_{i,j} = z_{i,j}(\ell_i, u_i)$ encodes the truncated component density $\tilde{f}_{j;\theta_j}$ from which x_i has been drawn. The respective conditional density of a generic complete observations of (X^t, Z) given $(L^t, U^t) = (\ell, u)$ becomes

$$\begin{aligned} f_{(X^t,Z)|(L^t=\ell,U^t=u)}(x, z) &= f_{X^t|(Z,L^t,U^t)=(z,\ell,u)}(x) \cdot \Pr(Z = z \mid L^t = \ell, U^t = u) \\ &= \Big(\prod_{j=1}^k \tilde{f}_{j;\theta_j}(x; \ell, u)^{z_j} \Big) \cdot \Big(\prod_{j=1}^k \tilde{p}_{j;p,\theta}(\ell, u)^{z_j} \Big). \end{aligned}$$

As a consequence, the complete sample weighted conditional likelihood is given by

$$\begin{aligned} &\ell(p, \theta | \mathfrak{C}) \\ &= \sum_{i=1}^N \sum_{j=1}^k w_i z_{i,j} \Big[\log \tilde{p}_{j;p,\theta}(\ell_i, u_i) + \log f_{j,\theta_j}(x_i) - \log F_{j;\theta_j}([\ell_i, u_i]) \Big] \\ &= \sum_{j=1}^k \sum_{i=1}^N w_i z_{i,j} \Big[\log p_j + \log f_{j;\theta_j}(x_i) - \log \Big(p_j F_{j;\theta_j}([\ell_i, u_i]) + \sum_{m \neq j} p_m F_{m;\theta_m}([\ell_i, u_i]) \Big) \Big], \end{aligned}$$

where $\mathfrak{C} = (x_i, \ell_i, u_i, w_i; z_{i,1}, \ldots, z_{i,k})_{i=1}^N$.

The E-step of the EM-algorithm now involves calculating the conditional expectation of the complete sample weighted conditional likelihood given the incomplete sample $\mathfrak{I} = (x_i, \ell_i, u_i, w_i)_{i=1}^N$. For that purpose, note that

$$\begin{aligned} P_j(x; p, \theta) &\equiv \Pr(Z_j = 1 \mid X^t = x, L^t = \ell, U^t = u) \\ &= \frac{\tilde{p}_{j;p,\theta}(\ell, u) \cdot \tilde{f}_{j;\theta_j}(x; \ell, u)}{\sum_{m=1}^k \tilde{p}_{m;p;\theta}(\ell, u) \cdot \tilde{f}_{m;\theta_m}(x; \ell, u)} = \frac{p_j \cdot f_{j;\theta_j}(x)}{\sum_{m=1}^k p_m \cdot f_{m;\theta_m}(x)} \end{aligned} \tag{S4}$$

where the last equation follows from (S3). Next suppose that, at the tth iteration of the algorithm, we are given an estimate $(p^{(t)}, \theta^{(t)})$. Then, under the assumption that $(p^{(t)}, \theta^{(t)})$ is the true parameter that generated $\mathfrak{C}$, the conditional expectation of $\ell(p, \theta|\mathfrak{C})$ given the incomplete sample $\mathfrak{I} = \mathfrak{I}_1 = (x_i, \ell_i, u_i)_{i=1}^N$ is given by

$$\begin{aligned} Q_{(p^{(t)},\theta^{(t)})}(p, \theta|\mathfrak{I}) &\equiv \mathbb{E}_{(p^{(t)},\theta^{(t)})}[\ell(p, \theta|\mathfrak{C}) \mid \mathfrak{I}] \\ &= \sum_{i=1}^N \sum_{j=1}^k w_i P_j(x_i; p^{(t)}, \theta^{(t)}) \Big[\log p_j + \log f_{j;\theta_j}(x_i) \end{aligned}$$

$$- \log\Big(p_j F_{j;\theta_j}([\ell_i, u_i]) + \sum_{m \neq j} p_m F_{m;\theta_m}([\ell_i, u_i])\Big)\Big], \tag{S5}$$

where we have used (S4). The M-step of the plain EM-algorithm [4] would now involve updating the parameter $(p^{(t)}, \theta^{(t)})$ by $(p^{(t+1)}, \theta^{(t+1)}) \in \arg\max_{(p,\theta)} Q_{(p^{(t)},\theta^{(t)})}(p, \theta|\mathfrak{I})$ and iterating until convergence. However, the M-step maximization problem is not feasible (which is essentially due to the random truncation), whence we propose to instead rely on a version of the EM-algorithm known as the ECME-algorithm [5]. The latter consists of dividing the M-step maximization problem into a series of $k+1$ lower-dimensional and feasible maximization problems (either 'ECM' or 'CM' steps), that are essentially based on successively maximizing $\theta_j \mapsto Q_{(p^{(t)},\theta^{(t)})}(p, \theta|\mathfrak{I})$ (ECM) and then $p \mapsto \ell(p, \theta|\mathfrak{I})$ (CM), holding all other parameters fixed:

Step 1. Maximize $Q_{(p^{(t)},\theta^{(t)})}(p, \theta|\mathfrak{I})$ subject to $g_1(p, \theta) = g_1(p^{(t)}, \theta^{(t)})$ where

$$g_1(p, \theta) = (p, (\theta_j)_{j \neq 1})$$

with solution $(p^{(t+1/(k+1))}, \theta^{(t+1/(k+1))})$.

Step 2. Maximize $Q_{(p^{(t)},\theta^{(t)})}(p, \theta|\mathfrak{I})$ subject to $g_2(p, \theta) = g_2(p^{(t+1/(k+1))}, \theta^{(t+1/(k+1))})$ where

$$g_2(p, \theta) = (p, (\theta_j)_{j \neq 2})$$

with solution $(p^{(t+2/(k+1))}, \theta^{(t+2/(k+1))})$.

$\vdots$

Step k. Maximize $Q_{(p^{(t)},\theta^{(t)})}(p, \theta|\mathfrak{I})$ subject to $g_k(p, \theta) = g_k(p^{(t+(k-1)/(k+1))}, \theta^{(t+(k-1)/(k+1))})$ where

$$g_k(p, \theta) = (p, (\theta_j)_{j \neq k})$$

with solution $(p^{(t+k/(k+1))}, \theta^{(t+k/(k+1))})$.

Step $k+1$. Maximize $\ell(p, \theta|\mathfrak{I})$ from (12) subject to $g_{k+1}(p, \theta) = g_{k+1}(p^{(t+k/(k+1))}, \theta^{(t+k/(k+1))})$ where

$$g_{k+1}(p, \theta) = \theta$$

with solution $(p^{(t+1)}, \theta^{(t+1)})$.

It can be shown that the space filling condition from Definition 2 in [6] is met (in that paper's notation we have $J_1(p, \theta) = \mathbb{R}^k \times \{0\} \times \mathbb{R}^{d_{\Theta_2}} \cdots \times \mathbb{R}^{d_{\Theta_k}}, \ldots, J_k(p, \theta) = \mathbb{R}^k \times \mathbb{R}^{d_{\Theta_1}} \times \cdots \times \mathbb{R}^{d_{\Theta_{k-1}}} \times \{0\}, J_{k+1}(p, \theta) = \{0\}^k \times \mathbb{R}^{d_\Theta}$, whence $J(p, \theta) = \bigcap_{j=1}^{k+1} J_j(p, \theta) = \{0\}$), and the algorithm is therefore known to converge to local maxima of $\ell(p, \theta|\mathfrak{I})$, given suitable regularity conditions.

Experimenting with the ECME-algorithm, we did in fact found a slight variant of the above algorithm that proved to converge more quickly in extensive computing experiments. The variant is based on modifying the jth step ($j = 1, \ldots, k$) described above as follows: instead of maximizing $(p, \theta) \mapsto Q_{(p^{(t)},\theta^{(t)})}(p, \theta|\mathfrak{I})$ subject to the given constraint, maximize

$$\theta_j \mapsto Q_{j;(p^{(t)},\theta^{(t)})}(\theta_j) = \sum_{i=1}^{N} w_i P_j(x_i; p^{(t)}, \theta^{(t)}) \Big[\log f_{j;\theta_j}(x_i) - \log F_{j;\theta_j}([\ell_i, u_i]) \Big]. \tag{S6}$$

Here, the change of the objective function may be motivated by rewriting

$$\begin{aligned} &Q_{(p^{(t)},\theta^{(t)})}(p, \theta|\mathfrak{I}) \\ &= \sum_{i=1}^{N} \sum_{j=1}^{k} w_i P_j(x_i; p^{(t)}, \theta^{(t)}) \Big[\log \tilde{p}_{j;p,\theta}(\ell_i, u_i) + \log f_{j,\theta_j}(x_i) - \log F_{j;\theta_j}([\ell_i, u_i]) \Big] \\ &\approx \sum_{i=1}^{N} \sum_{j=1}^{k} w_i P_j(x_i; p^{(t)}, \theta^{(t)}) \Big[\log \tilde{p}_{j;p^{(t)},\theta^{(t)}}(\ell_i, u_i) + \log f_{j,\theta_j}(x_i) - \log F_{j;\theta_j}([\ell_i, u_i]) \Big], \end{aligned}$$

and maximizing the latter subject to the given constraints is equivalent to maximizing (S6). Note that the approximation in the last display is typically quite accurate for large t, when the algorithm is close to convergence.

Calculating a maximum of the function in (S6) may involve a further algorithm depending on some starting value, say $\theta_{j,0}$. Following the notation in (13) from the main paper, we rewrite any solution of such an algorithm as $\mathrm{CML}(\mathcal{F}_j, \mathfrak{I}_j, \theta_{j,0})$, where $\mathcal{F}_j = \{f_{j;\theta_j} : \theta_j \in \Theta_j\}$ denotes the jth (untruncated) component family, where $\mathfrak{I}_j = \mathfrak{I}_j^{(t)} := \{(x_i, \ell_i, u_i, w_i P_j(x_i; p^{(t)}, \theta^{(t)}))\}$ denotes re-weighted truncated data, and where $\theta_{j,0}$ denotes a starting value. In view of this notation, the overall algorithm may be summarized as in Algorithm 1 in the paper.

Appendix C Derivation of the Erlang Mixture adapted ECME Algorithm

In this section we will derive the adaptations for fitting Erlang Mixtures in the setting of Section 3.2. Recall that Erlang Mixture families are given by $\mathcal{F} = \{\sum_{i=1}^{k} p_i \cdot \Gamma_{\alpha_i,\theta} : p \in (0,1)^k, \|p\|_1 = 1, \theta \in (0, \infty), \alpha \in \mathbb{N}^k, \alpha_1 < \ldots < \alpha_k\}$.

We will start by providing details on a suitable version of the ECME when treating the shape parameters $\alpha \in \mathbb{N}^k$ as fixed and known. For some given interval truncated sample $\mathfrak{I} = \{(x_i, \ell_i, u_i, w_i) | \ell_i \leq x_i \leq u_i\}$ the goal is to find $(\hat{p}, \hat{\theta}) = \arg\max_{p,\theta} \ell(p, \theta|\mathfrak{I}, \alpha)$, where ℓ is the weighted conditional log likelihood function from (12) with $f_{(p,\theta)} = \sum_{j=1}^{k} p_j d\Gamma_{\alpha_j,\theta}$ and $F_{(p,\theta)}$ the respective

cdf. For solving the maximization problem, we propose to use an ECME algorithm that involves two maximization steps within each iteration. For that purpose note that, following the ideas at the beginning of Appendix B, the adaption from (S6) becomes

$$Q_{(p^{(t)},\theta^{(t)})}(\theta) := \sum_{i=1}^{N}\sum_{j=1}^{k} w_i \cdot P_j(x_i; p^{(t)}, \theta^{(t)}) \Big[\log \mathrm{d}\Gamma_{\alpha_j,\theta}(x_i) - \log \Gamma_{\alpha_j,\theta}([\ell_i, u_i]) \Big].$$

Each iteration in the adapted ECME-Algorithm for Erlang Mixtures now consists of two steps:

Step 1. Maximize $Q_{(p^{(t)},\theta^{(t)})}(\theta)$ with solution $\theta^{(t+1/2)}$.

Step 2. Maximize $\ell(p, \theta|\mathfrak{I}, \alpha)$ subject to $g_2(p, \theta) = g_2(p^{(t)}, \theta^{(t+1/2)})$ where $g_2(p, \theta) = \theta$ with solution $(p^{(t+1)}, \theta^{(t+1)})$.

Note that the first step was decomposed into k separate steps when treating general mixtures that do not involve common parameters. The procedure is summarized in Algorithm S.2.

Algorithm S.2 Erlang Mixture ECME-Algorithm

function ErlangECME$(\mathcal{F}, \mathfrak{I}, p_0, \theta_0, \alpha, \varepsilon)$
  $p \leftarrow p_0$
  $\theta \leftarrow \theta_0$
  $l \leftarrow -\infty$
  repeat
    $l_0 \leftarrow l$
    $\theta' \leftarrow \arg\max_{\theta'} Q_{(p,\theta)}(\theta')$ ▷ Erlang ECM-Step
    $\theta \leftarrow \theta'$
    $p \leftarrow \arg\max_p \ell(p, \theta|\mathfrak{I}, \alpha)$ ▷ Erlang CM-Step
    $l \leftarrow \ell(p, \theta, \alpha|\mathfrak{I})$
  until $l - l_0 < \varepsilon$ ▷ Likelihood converged
end function

Algorithm S.2 requires starting values p_0 and θ_0 and a fixed shape parameter α_0, for which we found a K-Means approach that is partly similar to [7] to work well:

1. Run K-Means on the observed data $\{x : (x, \ell, u) \in \mathfrak{I}_1\}$ – ignoring truncation – with prescribed number of components k. Denote the obtained cluster centers by $c_1 < c_2 < \cdots < c_k$.
2. Estimate the preliminary scale parameter $\delta = \min_j\{c_j - c_{j-1}\}$ with $c_0 = 0$. This choice ensures that all clusters are separated enough to obtain pairwise different shape parameters in the next step.
3. Use $\alpha_{0,j} = \mathrm{round}(c_i/\delta), j = 1, \ldots, k$ as starting values for the shape parameters.
4. Set $m = \max_{(x,\ell,u,w)\in\mathfrak{I}} x/\alpha_{0,k}$ such that $(0, m\alpha_{0,k}]$ covers all observations.

5. Initialise the mixture weights $p_{0,j} = \#(\{(x, \ell, u) \in \mathfrak{I}_1 : \alpha_{0,j-1} m < x \leq \alpha_{0,j} m\})/N$ (where $\alpha_{0,0} := 0$) by tabulating, compare [8, Sec. 3.2].
6. Initialise θ_0 via moment matching – ignoring truncation, that is, let

$$\theta_0 = \frac{\bar{x}}{\sum_{j=1}^{k} p_{0;j} \alpha_{0,j}},$$

where $\bar{x} = \frac{1}{N} \sum_{(x,\ell,u,w) \in \mathfrak{I}} x$ is the observed mean value.

Finally, optimising the shape parameter essentially requires to solve an integer optimization problem. We propose to use a local shape search as in [8, Sec. 3.3]:

1. Compute a fit with starting values α_0, p_0, θ_0 as described above, and set $\alpha = \alpha_0$, with components $(\alpha_1, \ldots, \alpha_k)$.
2. Try fitting $(\alpha_1, \ldots, \alpha_{k-1}, \alpha_k + 1)$, and keep the new parameters if the log-likelihood improves, repeating until it no longer increases. Proceed increasing $\alpha_{k-1}, \ldots, \alpha_1$ one by one until no more improvements are found.
3. Try fitting $(\alpha_1 - 1, \alpha_2, \ldots, \alpha_k)$ and keep the new parameters if the log-likelihood improves, repeating until it no longer decreases. Proceed decreasing $\alpha_2, \ldots, \alpha_k$ one by one until no more improvements are found.
4. Go back to 2. if any improvement was found.

For steps 2. and 3. good starting values for p and θ are those from the currently selected parameters, since the potential changes in α are small for each step.

Appendix D True relationships in simulation

Throughout this section we provide details on the model specifications used within the simulation experiment in Section 5.

Baseline.

The following paragraph describes the relationships chosen for the baseline scenario. Other scenarios inherit their relationships from the baseline, changing only one relationship at a time.

The conditional claim feature distribution $P_Y(x,t) = P_Y(x)$ is defined in terms of the binary conditional distribution of `cc` and the conditional distribution of `severity`, conditioned on the variables shown:

$$\begin{aligned} P(\texttt{cc} = \text{material} | \texttt{ac}, \texttt{power}, \texttt{dens}) &= \operatorname{logit}^{-1}\big(0.5 + 0.05 \log(\texttt{dens}) - 0.1 \cdot \min(10, \texttt{ac}) \\ &\qquad - 0.05 \cdot \texttt{power} + 0.01 \cdot \min(10, \texttt{ac}) \cdot \texttt{power}\big), \\ &=: P^{\text{Baseline}}(\texttt{ac}, \texttt{power}, \texttt{dens}) \end{aligned}$$

$$P(\texttt{severity} \in \cdot | \texttt{cc}, \texttt{brand}, \texttt{ac}, \texttt{power}) = \log \mathcal{N}(\mu, \sigma),$$

where

$$\mu^{\text{Baseline}} := \mu = 5 + 0.35 \cdot \texttt{power} + 0.35 \cdot (2 - \texttt{ac})_+ - 0.05 \cdot \mathbf{1}(\texttt{brand} \in \{\text{B1}, \text{B2}, \text{B12}\})$$

$$+ 1.0 \cdot \mathbf{1}(\texttt{brand} \in \{\text{B10}, \text{B11}\})$$

$$\sigma^{\text{Baseline}} := \sigma = 9 - 0.01 \cdot (5 - \texttt{ac})_+ + 0.08 \cdot \texttt{power}.$$

The parametrization for the log-normal distribution is such that $X \sim \log \mathcal{N}(\mu, \sigma)$ means $\log X \sim \mathcal{N}(\mu, \sigma)$; in particular, $\mathbb{E}(X) = \exp(\mu + \sigma^2/2)$.

The reporting delay distribution $P_D(x, t, y) = P_D(x, y)$ is chosen as a BDEGP$(n = 1, m = 3, \kappa = 3 \cdot 365, \varepsilon = 365/2)$-distribution with parameters depending on $\texttt{dens}, \texttt{ac}, \texttt{cc}$ and $\texttt{severity}$ as follows: denoting

- p_0, the weight for δ_0
- p_1, the weight for $\Gamma(1, \theta)$
- p_2, the weight for $\Gamma(3, \theta)$
- p_3, the weight for $\Gamma(6, \theta)$
- p_4, the weight for GPD(κ, σ, ξ)
- θ, the common Erlang scale
- σ, the GPD scale
- ξ, the GPD shape

we set

$$\begin{aligned}
p_0 &= (1 - p_4) \operatorname{logit}^{-1}\Big(-4 - 0.5 \cdot \mathbf{1}(\texttt{cc} = \text{material}) + 0.5 \cdot \mathbf{1}(\texttt{ac} \leq 1 \wedge \texttt{cc} = \text{material}) \\
&\qquad - 0.25 \cdot \texttt{severity} \cdot \begin{cases} 10^{-3} & \texttt{cc} = \text{material} \\ 10^{-4} & \texttt{cc} = \text{injury} \end{cases} + 0.2 \cdot \min(1, |\texttt{age} - 45|/15)^2 \\
&\qquad + 0.01 \cdot \log(\texttt{dens})\Big) \\
&=: (1 - p_4) q_0^{\text{Baseline}} \\
p_1 &= (1 - p_4 - p_0) \cdot \operatorname{logit}^{-1}\Big(1 - 0.5 \cdot \mathbf{1}(\texttt{cc} = \text{material}) + \mathbf{1}(\texttt{ac} \leq 1 \wedge \texttt{cc} = \text{material}) \\
&\qquad - 2 \cdot \texttt{severity} \cdot \begin{cases} 10^{-3} & \texttt{cc} = \text{material} \\ 10^{-4} & \texttt{cc} = \text{injury} \end{cases} + 0.2 \cdot \min(1, 2 - \texttt{age}/25)_+\Big) \\
&=: (1 - p_4 - p_0) q_1^{\text{Baseline}} \\
p_2 &= (1 - p_4 - p_0 - p_1) \cdot \frac{p_1}{1 - p_4 - p_0} \\
p_3 &= 1 - p_4 - p_0 - p_1 - p_2 \\
p_4 &= \begin{cases} 0.0005 & \texttt{cc} = \text{material} \\ 0.02 & \texttt{cc} = \text{injury} \end{cases} \\
\theta &= \begin{cases} 30 & \texttt{cc} = \text{material} \\ 180 & \texttt{cc} = \text{injury} \end{cases} \\
\sigma &= \begin{cases} 180 & \texttt{cc} = \text{material} \\ 365 & \texttt{cc} = \text{injury} \end{cases} \\
\xi &= 0.2
\end{aligned}$$

Exposure Scenarios

There is a pool of 500,000 risks from the original dataset, risk features of policies created at time t are drawn uniformly from the pool. In the exposure scenario, this pool for new risks is downsampled for $\texttt{ac} \leq 5$ by a factor of $f(t) = 0.1 + 0.9 \cdot (1 - t/3650)$ (2a) and $f(t) = 0.1 + 0.9 \cdot \mathbf{1}(t \geq 3650/2)$ (2b). That means, policies created at time t are drawn uniformly from a modified pool consisting of all 244,230 original risks with $\texttt{ac} > 5$, and $f(t) \cdot 255,770$ risks with $\texttt{ac} \leq 5$ where the original dataset consists of 255,770 risks with $\texttt{ac} \leq 5$.

Claim Intensity Scenarios

The baseline claim intensity of $\lambda(x,t) = \texttt{truefreq}$ is reduced by 20% for all risks:

$$\lambda(x,t) = \texttt{truefreq} \cdot (1 - 0.2 \cdot t/3650), \tag{3a}$$
$$\lambda(x,t) = \texttt{truefreq} \cdot (1 - 0.2 \cdot \mathbf{1}(t \geq 3650/2)). \tag{3b}$$

Occurence Process Scenarios

The parameters for the claim feature distributions ($\texttt{cc}$ and $\texttt{severity}$) are changed depending on t: writing $p = P(\texttt{cc} = \text{material} | \texttt{ac}, \texttt{power}, \texttt{dens}, t)$, we have

$$\begin{cases} p = \text{logit}^{-1}\big(\text{logit}(P^{\text{Baseline}}(\texttt{ac}, \texttt{power}, \texttt{dens})) + 0.9t/3650\big), \\ \mu = \mu^{\text{Baseline}} + \begin{cases} 1.0 & \texttt{cc} = \text{material} \\ -0.5 & \texttt{cc} = \text{injury} \end{cases} \cdot t/3650, \\ \sigma = \sigma^{\text{Baseline}} + 0.5 \cdot \mathbf{1}(\texttt{cc} = \text{injury}) \cdot t/3650, \end{cases} \tag{4a}$$

$$\begin{cases} p = \text{logit}^{-1}\big(\text{logit}(P^{\text{Baseline}}(\texttt{ac}, \texttt{power}, \texttt{dens})) + 0.9 \cdot \mathbf{1}(t \geq 3650/2)\big), \\ \mu = \mu^{\text{Baseline}} + \begin{cases} 1.0 & \texttt{cc} = \text{material} \\ -0.5 & \texttt{cc} = \text{injury} \end{cases} \cdot \mathbf{1}(t \geq 3650/2), \\ \sigma = \sigma^{\text{Baseline}} + 0.5 \cdot \mathbf{1}(\texttt{cc} = \text{injury}, t \geq 3650/2). \end{cases} \tag{4b}$$

Reporting Process Scenarios

The baseline probabilities p_0 and p_1 are modified on the logit scale, keeping all other relationships in tact (i.e. p_4 does not change, p_2 and p_3 are defined via the modified p_0 and p_1 in the same way as in the baseline scenario):

$$\begin{cases} p_0 = (1 - p_4) \cdot \text{logit}^{-1}(\text{logit}(q_0^{\text{Baseline}}) + 2t/3650), \\ p_1 = (1 - p_4 - p_0) \cdot \text{logit}^{-1}(\text{logit}(q_1^{\text{Baseline}} + 2t/3650)), \end{cases} \tag{5a}$$

$$\begin{cases} p_0 = (1 - p_4) \cdot \text{logit}^{-1}(\text{logit}(q_0^{\text{Baseline}}) + 2 \cdot \mathbf{1}(t \geq 3650/2)), \\ p_1 = (1 - p_4 - p_0) \cdot \text{logit}^{-1}(\text{logit}(q_1^{\text{Baseline}} + 2 \cdot \mathbf{1}(t \geq 3650/2))). \end{cases} \tag{5b}$$

References

[1] Goodfellow, I.J., Bengio, Y., Courville, A.: Deep Learning. MIT Press, Cambridge, MA, USA (2016). http://www.deeplearningbook.org

[2] Ioffe, S., Szegedy, C.: Batch normalization: Accelerating deep network training by reducing internal covariate shift. In: Proceedings of the 32nd International Conference on International Conference on Machine Learning - Volume 37. ICML'15, pp. 448–456. JMLR.org, Lille, France (2015)

[3] Glorot, X., Bengio, Y.: Understanding the difficulty of training deep feedforward neural networks. In: Teh, Y.W., Titterington, M. (eds.) Proceedings of the Thirteenth International Conference on Artificial Intelligence and Statistics. Proceedings of Machine Learning Research, vol. 9, pp. 249–256. PMLR, Chia Laguna Resort, Sardinia, Italy (2010). http://proceedings.mlr.press/v9/glorot10a.html

[4] Dempster, A.P., Laird, N.M., Rubin, D.B.: Maximum likelihood from incomplete data via the em algorithm. Journal of the Royal Statistical Society. Series B (Methodological) **39**(1), 1–38 (1977)

[5] Liu, C., Rubin, D.B.: The ECME algorithm: a simple extension of EM and ECM with faster monotone convergence. Biometrika **81**(4), 633–648 (1994)

[6] Meng, X.-L., Rubin, D.B.: Maximum likelihood estimation via the ECM algorithm: A general framework. Biometrika **80**(2), 267–278 (1993)

[7] Gui, W., Rongtan, H., Lin, X.: Fitting the erlang mixture model to data via a gem-cmm algorithm. Journal of Computational and Applied Mathematics **343** (2018)

[8] Lee, S.C.K., Lin, X.S.: Modeling and evaluating insurance losses via mixtures of erlang distributions. North American Actuarial Journal **14**(1), 107–130 (2010)
