# Supplementary material for: Micro-level prediction of outstanding claim counts based on novel mixture models and neural networks
Source: Eur Actuar J. 2022 May 12;13(1):55–90. doi: 10.1007/s13385-022-00314-4 (PMC9098157; doi:10.1007/s13385-022-00314-4)
Supplement: Supplementary file 2 — Supplementary file2 (PDF 39 KB) [file 13385_2022_314_MOESM2_ESM.pdf]

Supplementary Material: Mirco-level Prediction of Outstanding Claim Counts using Neural Networks

Axel Bücher and Alexander Rosenstock

An R-File containing the functions used for the simulation study can be found on GitHub under the following link:

https://gist.github.com/AshesITR/d3023021e3e86ceb7ad661e91d5bc458
